# S100P enhances the motility and invasion of human trophoblast cell lines

**DOI:** 10.1038/s41598-018-29852-2

**Published:** 2018-07-31

**Authors:** Maral E. A. Tabrizi, Tara L. Lancaster, Thamir M. Ismail, Athina Georgiadou, Ankana Ganguly, Jayna J. Mistry, Keqing Wang, Philip S. Rudland, Shakil Ahmad, Stephane R. Gross

**Affiliations:** 1 School of Life and Health Sciences, Aston University, Birmingham, B4 7ET UK; 2 Institute of Integrative Biology, University of Liverpool, Biosciences Building, Crown Street, Liverpool, L69 7ZB UK; 3 Aston Medical Research Institute, Aston Medical School, Aston University, Birmingham, B4 7ET UK; 4 Institute of Metabolism and Systems Research, the University of Birmingham, Birmingham, B15 2TT UK

## Abstract

S100P has been shown to be a marker for carcinogenesis where its expression in solid tumours correlates with metastasis and a poor patient prognosis. This protein’s role in any physiological process is, however, unknown. Here we first show that S100P is expressed both in trophoblasts *in vivo* as well as in some corresponding cell lines in culture. We demonstrate that S100P is predominantly expressed during the early stage of placental formation with its highest expression levels occurring during the first trimester of gestation, particularly in the invading columns and anchoring villi. Using gain or loss of function studies through overexpression or knockdown of S100P expression respectively, our work shows that S100P stimulates both cell motility and cellular invasion in different trophoblastic and first trimester EVT cell lines. Interestingly, cell invasion was seen to be more dramatically affected than cell migration. Our results suggest that S100P may be acting as an important regulator of trophoblast invasion during placentation. This finding sheds new light on a hitherto uncharacterized molecular mechanism which may, in turn, lead to the identification of novel targets that may explain why significant numbers of confirmed human pregnancies suffer complications through poor placental implantation.

## Introduction

Trophoblast invasion of the decidualised endometrium to establish the precursor of the placenta, the first step of implantation, is a tightly regulated process, orchestrated by the continuous cross-talk between foetal and maternal compartments. During this stage, one of the prominent factors for proper embryonic development is the successful migration and invasion of extravillous trophoblast cells into the maternal decidua and myometrium. Shallow implantation, in contrast, is thought to lead to poor blood and nutrient supplies to the developing foetus, ultimately resulting in pregnancy conditions such as foetal growth restriction, preeclampsia and miscarriages.

A class of proteins that has been linked to the process of placentation development and pregnancy disorders is the S100 family of calcium-binding proteins. This family of approximately 25 different proteins is characterised by the presence of a pair of calcium-binding helix-loop helix domains (EF hand regions) at either end of the protein sequences. Whilst these proteins do not contain intrinsic enzymatic activities of their own, their interaction with specific partners regulates a large number of cellular components and biological processes both intracellularly and extracellularly. For instance, upregulation of both S100A6 and S100A12 has been linked to increases in preeclampsia^1,2^. Expression of other S100 proteins in the process of placentation has been reported with the majority concentrated on this expression on the maternal/endometrial sides, where, for instance S100G (also known as Calbindin-d9k)^3^, CaBP-d28k^4^, S100A10^5^ and S100A11^6^ have been linked to regulating endometrial receptivity. Reports of occurrence of S100 protein expression from the foetal side have been more infrequent, although for example, CaBP-d28k has been reported to be expressed in trophoblast Jeg-3 cells^7^ and *in vivo*^8^, whilst S100B, S100A4, S100A6 and S100A8 expression have been observed in trophoblasts^9–12^. However the true functions of S100 proteins in either the endometrium or trophoblasts remain unknown.

Another S100 factor, S100P has been shown to be expressed in the placenta^13,14^ and is thought to play important roles during placentation, where it is found at a concentration 90–200-fold higher than in any other organs^15^. Its expression also occurs in the endometrial tissue, predominantly in the endometrial epithelial and stromal cells, where its increasing levels correlate with those of P4 progesterone upon stimulation^16^. Its expression is also dramatically upregulated during the formation of the receptive window, an observation that appeared to be S100P specific, since only minor changes were seen with other S100 proteins studied^17^. S100P has also recently been reported to be expressed in trophoblasts^18–20^, but the role of this protein in the implantation process remains obscure.

Our work in this report demonstrates that S100P can stimulate the motile and invasive properties of trophoblasts. We show that this protein is greatly expressed in human trophoblasts *in vivo*. Expression is seen in the extravillous populations of the anchoring columns, with an overall expression seen to be at its highest during the early stages of implantation in the first trimester of gestation. High levels of S100P expression can equally be observed in two human placental-derived trophoblastic cell lines Jeg-3 and Bewo. Interestingly high levels of S100P expression promote both motility and invasion in these lines since its specific down-regulation using targeted siRNA technology is sufficient to abrogate these properties. Cellular analysis also demonstrates that reduced levels of S100P leads to dramatic changes in cytoskeletal remodelling. The reciprocal experiment, using the first trimester HTR8/SVneo extravillous trophoblast cell line, in which S100P expression is overexpressed, equally demonstrates that high levels of S100P correlates with significant increases in motility and invasion. Our data therefore demonstrates a new function for S100P which links its well characterised motility and invasion- enhancing properties to a physiological process, that of placental implantation.

## Material and Methods

### Human Placental tissues

Written informed consent was obtained from all women recruited into the study. Samples of placenta tissues obtained immediately after elective termination of pregnancy from first trimester of gestation (8–11 weeks; n = 5), second trimester (15–20 weeks; n = 4), or third trimester (32–38 weeks; n = 7) were collected using mifepristone and misoprostol or from pregnancies delivered by elective Caesarean with tissues processed as described previously^21^. Placental samples were collected with approval of Health Research Authority - West Midlands, Edgbaston Research Ethics Committee (NHS REC 06/Q2707/12 [2006 approval]) (RG_14–194 [10.2014 approval]) and by the Lothian Research Ethics Committee (NHS REC 09/S0704/3). Samples were fixed with formaldehyde and embedded into paraffin wax prior to further processing. For all samples, written informed consent was obtained and all methods were performed in accordance with the NHS and HRA guidelines.

### Cell Lines and culture

The two human placental derived choriocarcinoma trophoblastic cell lines Jeg-3 and Bewo were a kind gift of Dr. Emmanouil Karteris (Brunel University). The HTR8/SVneo first trimester extravillous (EVT) trophoblasts were a gift of Prof. Graham Charles (Queen’s University, Kingston, Ontario (Canada)) These cell lines are some of the best characterised lines to study motility and invasion in respect to implantation/placentation^22^. The human cancer cell line HeLa A3 expressing the S100P protein under the control of a doxycycline inducible promoter has been reported previously^23^. Cells were cultured with 5% (v/v) CO_2_ and 20% (v/v) O_2_ at 37 °C in MEM (for Jeg-3), F-12 medium (for Bewo), RPMI (HTR8/SVneo) and normal DMEM (for HeLa), supplemented with 10% (v/v) Fetal Bovine Serum (FBS), 100 units and 0.1 mg/ml Penicillin/streptomycin respectively and 2mM L-glutamine. Trophoblast cells were passaged using 0.025% (w/v) trypsin in 2.5 mM EDTA and all experiments were conducted with cells within 10–15 passages. The HeLa A3 inducible cells were grown, induced and treated as previously described^23^. In the context of our experiments, using HeLa A3 cells as both positive and negative controls, induction was promoted for 48–72 hours with 1.5 μg/ml doxycycline addition.

### Stably expressing S100P HTR8/SVneo cell lines

The HTR8/SVneo cells were grown on 6 well plate to 70–80% confluency at the time of transfection (500,000 cells/well) in RPMI 1640 medium supplemented with 5% (v/v) FCS for 24 hours prior to transfection with SGB217 with Lipofectamine 3000 (Invitrogen) following manufacturers’ instructions and the clones selected using 50 μg/ml hygromycin B. Plasmid SGB217 was obtained by *in vivo* recombination of pcDNA3.1 Hygro plasmid (ThermoFisher, UK) with a PCR amplified S100P product using SLiCE (Seamless ligation cloning extract). The successfully growing clones were isolated and transferred to 24 well plate to grow up separately in medium with hygromycin B before expansion and further characterisation.

### siRNA S100P and control delivery

Cells seeded (30000 Jeg3 cells and 60000 Bewo cells) in 24 well plates were grown for 2 days (Jeg-3) or 5 days (Bewo) prior to being transfected with 5 nM double-stranded siRNA (Qiagen, UK) for S100P (siRNA 4: SI00709940 and siRNA 6: SI03247013;) or with a mock control siRNA (SI03650318) in OptiMEM (Gibco, UK) and normal medium using 2 μl/well INTERFERin transfection reagents (Polyplus, France) following the manufacturer’s instructions. Cells were left in the presence of the different siRNAs for 48 hours prior to collection for qPCR or 72 hours for Western blotting analysis (See below). For motility/invasion and immunostaining, cells were left to grow for 48 hours prior to starting the experiment.

### Cell counting and viability using trypan blue exclusion

Cells treated with different siRNA (as above) or HTR8 cells were seeded at a density of 20000 cells per well in 24 well plates and grow for a further 24–48 hours incubation. At each respective time point, cells were removed using 0.025% (w/v) trypsin in 2.5 mM EDTA, diluted in serum-containing medium before centrifuging and resuspension in a mixture of 100 μl PBS/trypan blue. Cell numbers were calculated by haemocytometer counting and established as number of cells/well. Data is presented as percentage means ± SD of 3 independent experiments relative to controls over the course of the study.

### Western blotting

Following siRNA treatment for 72 hours, cells were collected by scrapping in 1 × PBS with 1x cocktail protease inhibitors (Sigma, UK) prior to sonication and dilution in 5x Laemmli buffer^24^. Protein lysates (25 μg) were loaded onto 16% (w/v) polyacrylamide gels and transferred to PVDF membranes with 30 mA per blot for 2 hours prior to blocking in blocking buffer (3% (w/v) BSA in PBS). Polyclonal goat anti S100P (R&D, UK) or monoclonal mouse/rabbit antibodies to α-tubulin and β-actin (Abcam, UK) antibodies were diluted in blocking buffer (Supplementary Table S1) and incubated overnight at 4 °C, prior to washing and incubation with the relevant secondary antibodies conjugated to HRP and ECL development (anti-goat or anti-rabbit/mouse (Supplementary Table S1)). For quantification purposes, levels of S100P were measured by densitometry analysis after Western blotting and normalised to the housekeeping proteins β-actin or α-tubulin for all samples. Densitometry quantification are presented as means ± SD of 3 independent experiments.

### qPCR analysis

Following the appropriate incubation, trophoblast cells were collected after trypsinisation and mRNA extraction was carried out using TRIS reagent (Sigma, UK) according to the manufacturer’s protocol. qPCR was performed using S100P (sequences F: 5′-TCAAGGTGCTGATGGAGAA-3′ and R: 5′-ACACGATGAACTCACTGAA-3′) and β-actin primers (F: 5′-ATGTACGTTGCTATCCAGGC-3′ and R: 5′-CTCCTTAATGTCACGCACGAT-3′) and SYBR green mix (Roche, UK) according to the manufacturer’s protocols. Data of an individual representative experiment is presented as the mean values ± SD of 3 independent samples relative to controls.

### Immunofluorescent staining

Immunofluorescence was carried out as previously described^25^. Briefly trophoblast-derived cell lines Bewo and Jeg-3 cells either untreated or 48 hours following siRNA treatments were plated at a concentration of 25,000 cells/well onto fibronectin-coated (2.5 μg/cm²) glass coverslips in a 24-well plate. After a 24 hour incubation, cells were washed once in cytoskeleton buffer (CB: 150 mM NaCl, 5 mM MgCl_2_, 5 mM EGTA, 5 mM glucose, 10 mM 2-(N-morpholino) ethanesulfonic acid, pH 6.1) and fixed with 3.7% (w/v) paraformaldehyde in CB at 37 °C for 20 min followed by permeabilisation with 5% (v/v) Triton X-100 in CB for 2 min and blocking with blocking solution (5% (v/v) goat serum in CB) for 60 min. Primary antibody incubation (Supplementary Table S1) against S100P and paxillin in blocking solution (1% (v/v) goat serum in CB) was incubated for 45 min at 37 °C. After washing three times with 1% (v/v) goat serum in CB, cells were incubated with the appropriate secondary anti-rabbit or anti- mouse antibodies labelled with FITC or TRITC (Supplementary Table S1) respectively in blocking solution for 45 min at 37 °C. For actin staining, rhodamine phalloidin (Invitrogen, Paisley, UK) was also added with secondary antibodies at a concentration of 0.6 μM. After washing with blocking solution, coverslips were rinsed once with water and mounted in Vectashield mounting medium (Vector Labs, UK), before being viewed using an Epifluorescence Leica DMI400B microscope equipped with a 63x oil objective. Images of an individual representative experiment are presented. Quantification of focal adhesions was carried out by counting the number of focal adhesions per cell selected at random and are presented as means ± SD of 3 independent experiments.

For immunofluorescent staining of the wound assay, Jeg-3 either non-treated or 48 hours following siRNA treatments, were plated at a concentration of 50,000 cells/well onto fibronectin-coated (2.5 μg/cm²) glass coverslips in a 24-well plate and grown for 48 hours. The confluent monolayers were scored with a sterile pipette tip to leave a scratch of approximately 0.4–0.5 mm in width. Culture medium was removed and replaced with fresh medium. Cells were left to grow for a further 16 hours prior to staining.

### Immunohistochemistry

Immunohistochemical staining for S100P and counterstaining were performed simultaneously on human placental tissues as previously described^26^. All procedures and counterstaining for the groups were undertaken on the same day using the same reagents. Whenever required, staining was also performed on serial sections for multi-staining procedures. Tissue sections were deparaffinised, and heat-induced antigen-retrieval were performed in citrate buffer (pH 6.0) using a pressure cooker (Prestige Medical, UK). Non-specific protein binding was blocked by incubation with either 1% (v/v) normal bovine serum and 5% (v/v) goat IgG (Vector Labs, UK) or 10% (v/v) normal swine serum (Vector Labs, UK) for 1 hour and sections were then incubated with a goat polyclonal anti-S100P antibody (R&D, UK), a rabbit monoclonal anti-S100P antibody (Abcam, UK), a mouse monoclonal anti-HLA-G antibody (Abcam, UK), a rabbit polyclonal anti-CD49f (integrin α6) antibody (Abcam, UK) or an mouse monoclonal anti cytokeratin7 antibody (Leica Biosystems, UK) at 4 °C overnight (Supplementary Table S1). After washing in 0.1% (v/v) Tween 20 (Sigma-Aldrich, UK) in Tris buffered Saline pH 7.4, sections were then incubated with the appropriate second antibody conjugated to horseradish peroxidase (HRP, Supplementary Table S1). The staining was analyzed using a Nano Zoomer XR scanner (Hamamatsu) and image acquisition using NDP software. Image analysis was conducted using Image-J analysis software. Images of an individual representative experiment are presented.

### Motility/invasion assay

Motility and invasion abilities were measured using 8 μms Boyden polycarbonate transwell membranes (Greiner, UK). Following siRNA treatment for 48 hours and serum deprivation by growing the cells in 0.5% (v/v) FBS-containing medium for a further 24 hours, 10^5^ cells were seeded in 0.5% (v/v/) FBS containing medium on without (motility) or with congealed matrigel (1:3 with serum free media Sigma, UK) against a gradient of 10% (v/v) FBS medium in the outer wells. Cells were left to migrate through the membranes for 24 hours prior to fixing and staining using the Diffquik histochemical kit (Reagena, Finland) following the manufacturer’s instructions.

Stained cells on the upper surface of the membrane were removed and those on the lower side of the membrane were counted using a light microscope with an x20 objective lens, selecting 5 random fields. Data is presented as the means ± SEM of 3 or 4 independent experiments relative to controls (percentage) from 4 replicate wells for each set of conditions (Total cell numbers counted are available in Supplementary Tables S2 and S3). Images of representative fields of motility/invasion assays were taken with the EVOS XL Cell Imaging System at x20 magnification.

### Chemotaxis assay

Migration and chemotaxis abilities were measured using the Dunn chamber motility assay in DC100 Hawksley chambers. Following siRNA treatment for 48 hours and serum-deprivation in 0.5% (v/v) FBS-containing medium for a further 24 hours, 10^5^ cells were seeded in 0.5% (v/v) FBS-containing medium for a minimum of 5 hours on pre-coated fibronectin coverslips. Coverslips and chambers were setup according to previously published protocols^27^ prior to incubation in Cellstar 4 well plates (Greiner, UK). Digitised images were recorded using the Cell IQ automated image capture system, (Chip-Man Technologies, Finland) in which pre-selected fields were imaged using phase-contrast microscopy on a continuous loop for 24 hours. Quantification of migration and chemotaxis of an individual representative experiment was analysed by tracking of single cells (n = 20) using Image J with chemotaxis and migration tool plugins.

### Deparaffinisation of embedded placental tissues for protein lysate preparation

Samples were processed using the protocol for protein extraction from formalin-fixed paraffin embedded specimens^28^. Briefly, 5 tissues sections (10 μm each) were deparaffinised with a mixture of octane and methanol prior to drying and resuspension in 20 mM Tris-HCl buffer (pH 7) containing 2% (w/v) SDS prior to protein extraction using a 1 mL syringe. Protein quantification was performed using the BCA assay (Pierce).

### Statistical analysis

Results were analysed by one-way ANOVA followed by Tukey’s multiple comparison test using GraphPad Prism 7.03 (GraphPad Software, San Diego, CA). Results are presented as the means ± SD or SEM. Means are considered significantly different from each other are indicated by P < 0.05, P < 0.01 or P < 0.001.

## Results

### S100P is expressed in human trophoblasts including anchoring columns *in vivo*

**Figure 1:**
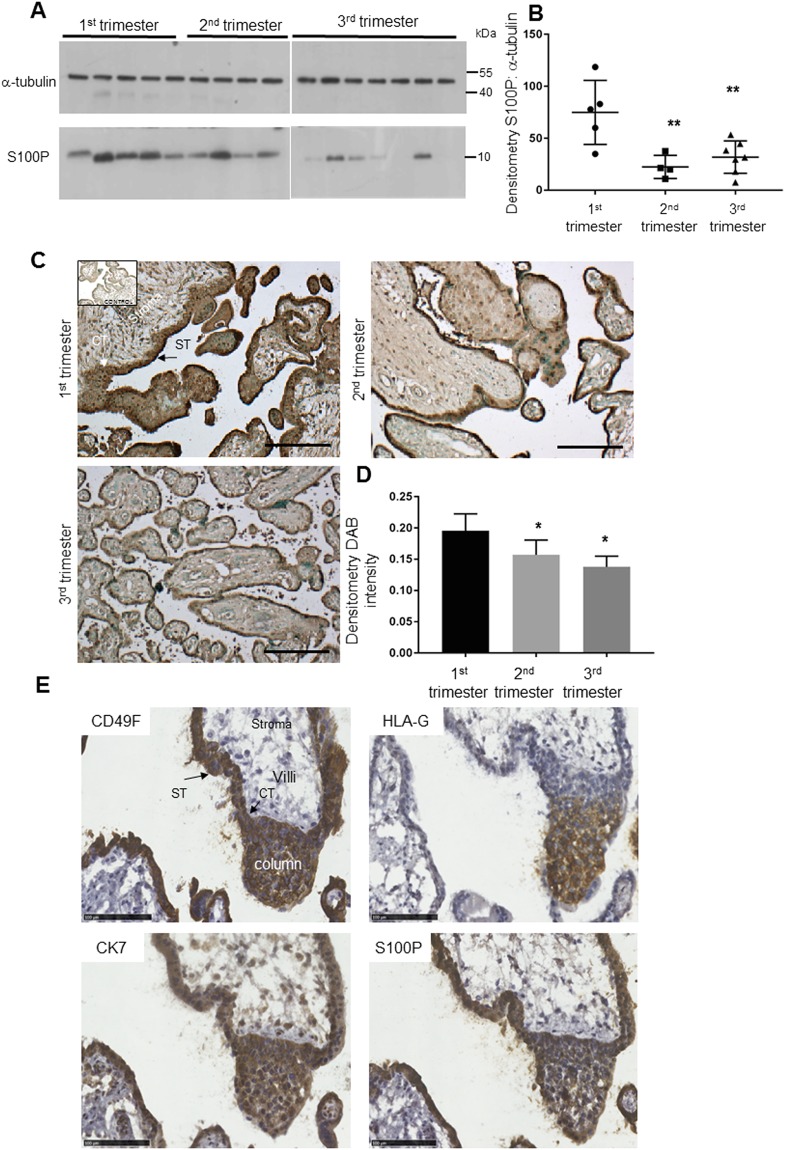
S100P is expressed in trophoblasts including extravillous trophoblasts and more significantly during the first trimester in placental tissues. Expression of S100P proteins was analysed on both lysates (**A**,**B**) and sections (**C**,**E**) obtained from different paraffin-embedded placental samples from different gestational periods (first trimester (n = 5), second trimester (n = 4), or third trimester (n = 7). Proteins extracted from paraffin-embedded placental block sections (1^st^ trimester; 2^nd^ trimester and 3^rd^ trimester) at equal loading were separated by SDS-PAGE electrophoresis. Western blotting was carried out and membranes probed for S100P or α-tubulin and cropped images are presented (**A**). Levels of S100P were measured by densitometry analysis after Western blotting and normalised to α-tubulin for all samples (first trimester (n = 5), second trimester (n = 4), or third trimester (n = 7). Data is presented as percentage means ± SD of 2 independent experiments compared to the first trimester (**B**). Immunohistochemistry staining using a goat polyclonal S100P antibody and counterstaining on human placental tissues was performed as described in Methods. Arrows indicate cytotrophoblast cells (CT), syncytium (ST). Stroma is also highlighted. Bar corresponds to 150 μm (**C**). Quantification of S100P DAB staining and intensity in 1^st^, 2^nd^ and 3^rd^ trimester serial sections. Data of an individual representative experiment is presented as the mean values ± SD of 3 independent samples (**D**). Statistical analysis (**B**,**D**) show ± SD compared to the first trimester samples of an individual representative experiment. *P < 0.05 and **P < 0.01 (one way- ANOVA). Immunohistochemistry staining using a panel of trophoblast marker antibodies (cytokeratin 7 (CK7), HLA-G, integrin α6 (CD49F) and S100P antibody) and counterstaining on anchoring columns of serial human placental tissues was performed as described in Methods. Arrows indicate cytotrophoblast cells (CT), syncytium (ST). Stroma is also highlighted. Bar corresponds to 100 μm (**E**).

Different reports have aimed to establish the expression patterns of S100P in human tissues^15,29^; its highest expression is found in placenta, from which it was originally isolated and characterised^13,14^. Little, however, is known about the cell types that express S100P within the placenta as well as its overall levels during the different stages of gestation. Human embryonic placenta tissues obtained immediately after elective termination of pregnancy at different times of gestation were prepared and embedded in paraffin wax. The protein lysates from the different formalin-fixed paraffin-embedded tissue sections were subsequently extracted, as previously described^28^, before protein levels were compared by SDS-PAGE and Western-blotting using antibodies to S100P and α-tubulin (Fig. 1A; full length blots are presented in Supplementary Fig. S2), and subsequent densitometric analysis performed (Fig. 1B) as well as following density analysis after immunohistochemistry of the different sections (Fig. 1C,D). The results show a reduction in levels of S100P as gestation progresses with levels reaching only 45% in the third trimester. (Fig. 1A,B,D). The decrease in S100P levels was seen to be significant throughout the gestational period between first trimester and second trimester (P = 0.0063 for Western blot analysis and P = 0.0287 for immunohistochemistry quantification) or third trimester (P = 0.095 for Western blot analysis and P = 0.0016 for immunohistochemistry quantification) but not between second and third trimester samples (P = 0.7568 for Western blot analysis and P = 0.3479 for immunohistochemistry quantification). This data suggests that S100P is predominantly highly expressed in the early stage of gestation, during the process of trophoblast implantation.

In order to determine the pattern of S100P expression in the different cell types of the placental tissues, paraffin-embedded sections from early gestational stages were stained for S100P, along with other different trophoblast markers, using immunohistochemistry techniques (Fig. 1C,E). We found that S100P was expressed in both human villous cytotrophoblasts and multinuclear syncytiotrophoblast structures of the placenta during the different stages of pregnancy (Fig. 1C). We also sought to investigate whether S100P expression was also present in the differentiating extravillous trophoblast populations in anchoring trophoblast columns (Fig. 1E). A strong S100P signal was observed within the extravillous trophoblast anchoring columns. Serial sections of placental villi stained for cytokeratin-7 and HLA-G confirmed the identity of the extravillous trophoblasts (Fig. 1E) and the presence of S100P expression at all proximal, middle and distal ends of the anchoring columns. Serially sectioned samples were also tested for antibody specificity by staining with the respective secondary antibodies alone (Supplementary Fig. S1).

Taken together, these results show that the S100P protein is predominantly expressed in the trophoblasts cells, including the extravillous trophoblast subsets and their anchoring columns and that S100P levels appear to be highest at times when trophoblasts are the most invasive during the early stages of placental implantation.

### S100P is expressed in human trophoblast Jeg-3 and Bewo cell lines but not in the cultured first trimester extravillous HTR8/SVneo cells

**Figure 2:**
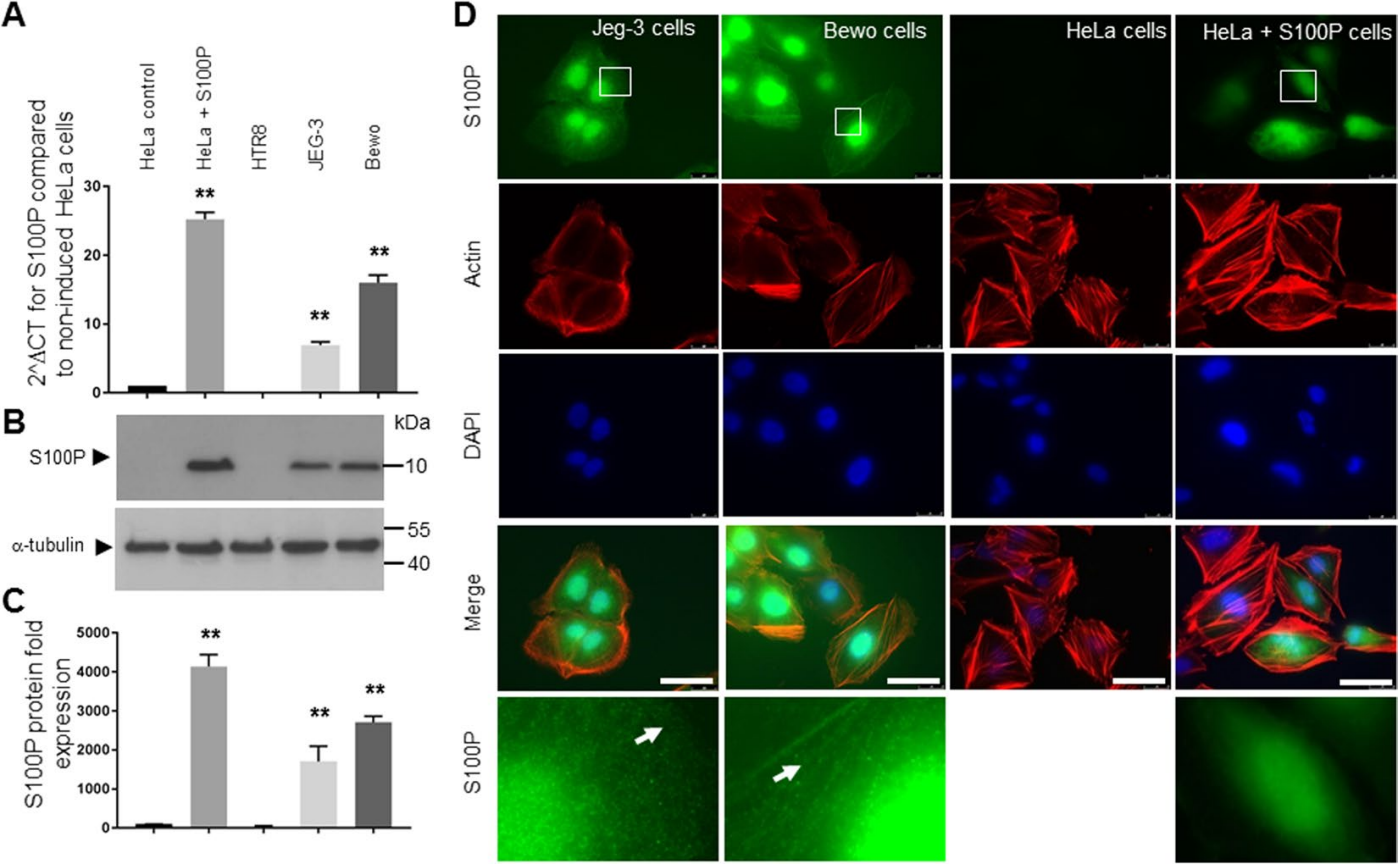
S100P is expressed in Jeg-3 and Bewo but not HTR8 EV trophoblast cell lines. HTR8, Bewo and Jeg-3 cells, along with HeLa A3 induced for S100P expression (or their non-expressing counterparts), were grown for 48 hours prior to collection for mRNA qPCR analysis (**A**) or 72 hours prior to collection for protein Western blotting (**B**). mRNAs were isolated using TRIS reagent followed by reverse transcription and quantitative PCR analysis using S100P and β-actin primers, as described in Methods. Data is presented as 2^∆^CT mean values ± SD of 3 independent samples of a representative experiment compared to the non-induced HeLaA3 cells. **P < 0.0001 (one way- ANOVA). (**A**) For the protein levels, cells were collected and solubilised in Laemmeli buffer and equal loading were separated by SDS-PAGE electrophoresis. Western blotting was carried out and membranes probed for S100P or α-tubulin and cropped images are presented. (**B**) Expression levels of S100P were measured by densitometric analysis, normalised to α-tubulin and presented in comparison to the non-induced HeLa A3 cells as percentage mean values ± SD of 3 independent samples compared to the non-induced HeLa A3 cells. **P < 0.0001 (one way- ANOVA). (**C**) For immunostaining, Bewo and Jeg-3 cells, along with HeLa A3 induced for S100P expression (or their non-expressing counterparts) were seeded on fibronectin-coated coverslips and grown for 48 hours prior to fixation, permeabilisation and staining for S100P and actin. Cells were mounted and viewed using epifluorescence microscopy. (**D**) Images in the last row correspond to the focused regions of the highlighted cells. Bar corresponds to 25 μm.

Having established that S100P is predominantly expressed in human trophoblastic cells in placental tissues, we sought to determine if its mRNA/protein expression could equally be observed in trophoblast cell lines in order to provide a tractable experimental system. HTR8/SVneo, BeWo and Jeg-3 cells have been shown to be invaluable lines to study trophoblastic functions in cultures^22,30^. Expression analysis for S100P in these lines, along with an S100P doxycycline-inducible HeLa A3 cell system (acting as both positive and negative controls^23^) was carried out by mRNA quantification (qPCR amplification, Fig. 2A) and by Western blotting for proteins (Fig. 2B; full length blots are presented in Supplementary Fig. S3). Whilst non-induced HeLa cells, acting as negative control, did not show any detectable levels of S100P by Western blotting (Fig. 2B) and only low background levels after qPCR, induced HeLa cells showed a 25–30 fold highly significant increase in the amount of S100P when quantifying either mRNA or protein. Interestingly, both Jeg-3 and Bewo cells also presented significant levels of endogenously expressed S100P (P < 0.0001 compared to non-induced HeLa), corresponding to about half of those seen in the S100P-inducible system, as determined by either densitometric analysis or by calculating the 2^∆^CT (Fig. 2). When comparing the two different trophoblastic cell lines, it was found that significantly higher levels of 1.7 to 2 fold were seen in the Bewo line when compared to the Jeg-3 counterparts. Surprisingly, we were not able to detect any expression of S100P by either Western blotting or qPCR in the first trimester extravillous HTR8/SVneo trophoblast cell lines (Fig. 2A,B) with levels observed being even lower than in the HeLa non-induced cells.

In order to learn more about S100P cellular localisation in the different trophoblast cells, immunostaining was carried out together with staining for both actin and the nucleus (Fig. 2D). To first confirm the specificity of the staining, we sought to stain for the S100P protein in our inducible HeLa system. As expected, there were no detectable levels of fluorescence against S100P in the cells grown without doxycycline. Induction of S100P in the HeLa cells resulted in an increase in fluorescence with staining observed in both the nuclear and cytoplasmic regions of the cells, consistent with previous work related to S100P localisation in these cell lines^23^. The majority of S100P in Jeg-3 and Bewo cells was found in a nuclear and perinuclear location, as observed by its colocalisation with DAPI, but some was also seen in the cytoplasmic compartment (Fig. 2D white arrow). This result indicates that S100P may be localised both in the nucleus and cytoplasm of trophoblast cell lineages. Antibody specificity was determined by staining with the corresponding secondary FITC labelled anti-IgG antibody alone, resulting in very weak background levels (data not shown). This data demonstrates that detectable levels of endogenous S100P are expressed in some trophoblast cell lines and that both Jeg-3 and Bewo cells can be used as models to silence S100P expression, but that S100P levels need to be overexpressed in the HTR8/SVneo cells since none are detectable.

### Knock down of S100P by siRNA in human trophoblasts Jeg-3 and Bewo cell lines

**Figure 3:**
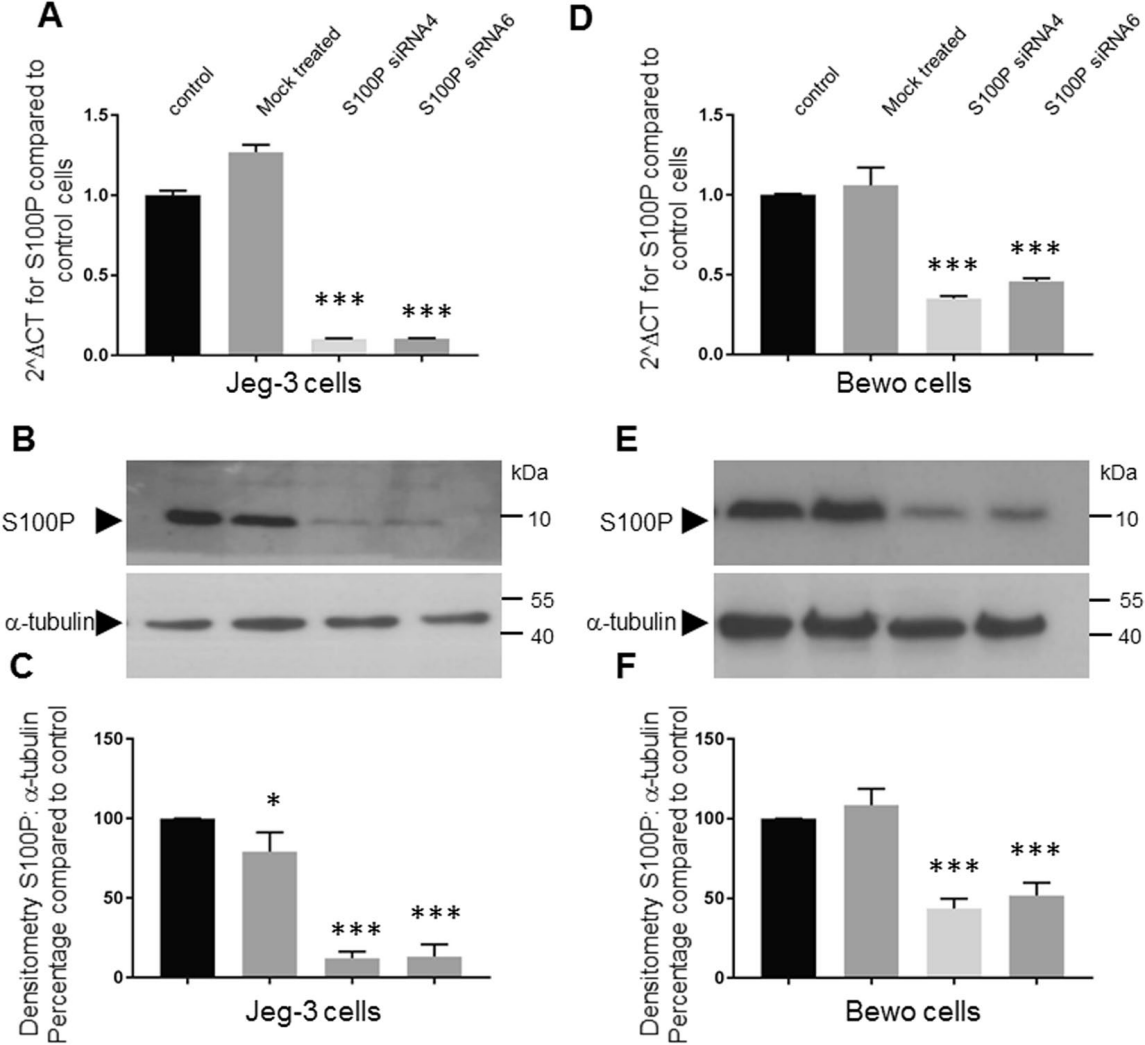
Specific knock-down of S100P in Jeg-3 and Bewo trophoblastic cell lines. Bewo and Jeg-3 cells were incubated in the presence of different S100P or control siRNAs for 48 hours prior to collection for mRNA qPCR analysis (**A**,**D**) or 72 hours prior to collection for protein Western blotting (**B**,**C**,**E**,**F**). mRNAs were isolated using TRIS reagent followed by reverse transcription and quantitative PCR analysis using primers for S100P and β-actin, as indicated in Methods. Data is presented as 2^∆^CT mean values ± SD of 3 independent samples of a representative experiment compared to non-treated control samples. ***P < 0.0001 (one way- ANOVA) (**A**,**D**). For protein levels, cells were collected and solubilised in Laemmeli buffer and equal loading were separated by SDS-PAGE electrophoresis. Western blotting was carried out and membranes probed for S100P or α-tubulin and cropped images are presented (**B**,**E**). Expression levels of S100P were measured by densitometric analysis, normalised to α-tubulin and presented as percentage mean values ± SD of 3 independent samples of a representative experiment compared to non-treated control samples. *P < 0.01 ***P < 0.0001 (one way- ANOVA) (**C**,**F**).

Having demonstrated the endogenous expression of S100P in Jeg-3 and Bewo trophoblast cell lines, we next wanted to determine if we could specifically down-regulate S100P levels by siRNA delivery in order to gain further understanding about its physiological roles. Two different siRNAs were tested to determine their efficacy in reducing S100P expression at both the mRNA level after 48 hours (Fig. 3A,D) and protein level 72 hours after treatment (Fig. 3B,C,E,F; full length blots are presented in Supplementary Fig. S4). Mock treatment did not result in any appreciable changes in the levels of S100P expression in either Jeg-3 or Bewo cells. However, the presence of S100P-targeted siRNA always resulted in a significant reduction of S100P levels, albeit with different effectiveness. In general S100P directed siRNA treatments were less effective in Bewo cells, leading to a maximum reduction of 2.5 fold or 50–60% decrease in S100P expression compared to 9.7 fold or over 90% decrease in S100P expression in the Jeg-3 cells. Although a total silencing of S100P expression was not achieved, significant reductions in S100P levels were still observed, especially in the Jeg-3 lineage.

### Knock-down of S100P results in significant reductions in Jeg-3 and Bewo cell motility and migration

**Figure 4:**
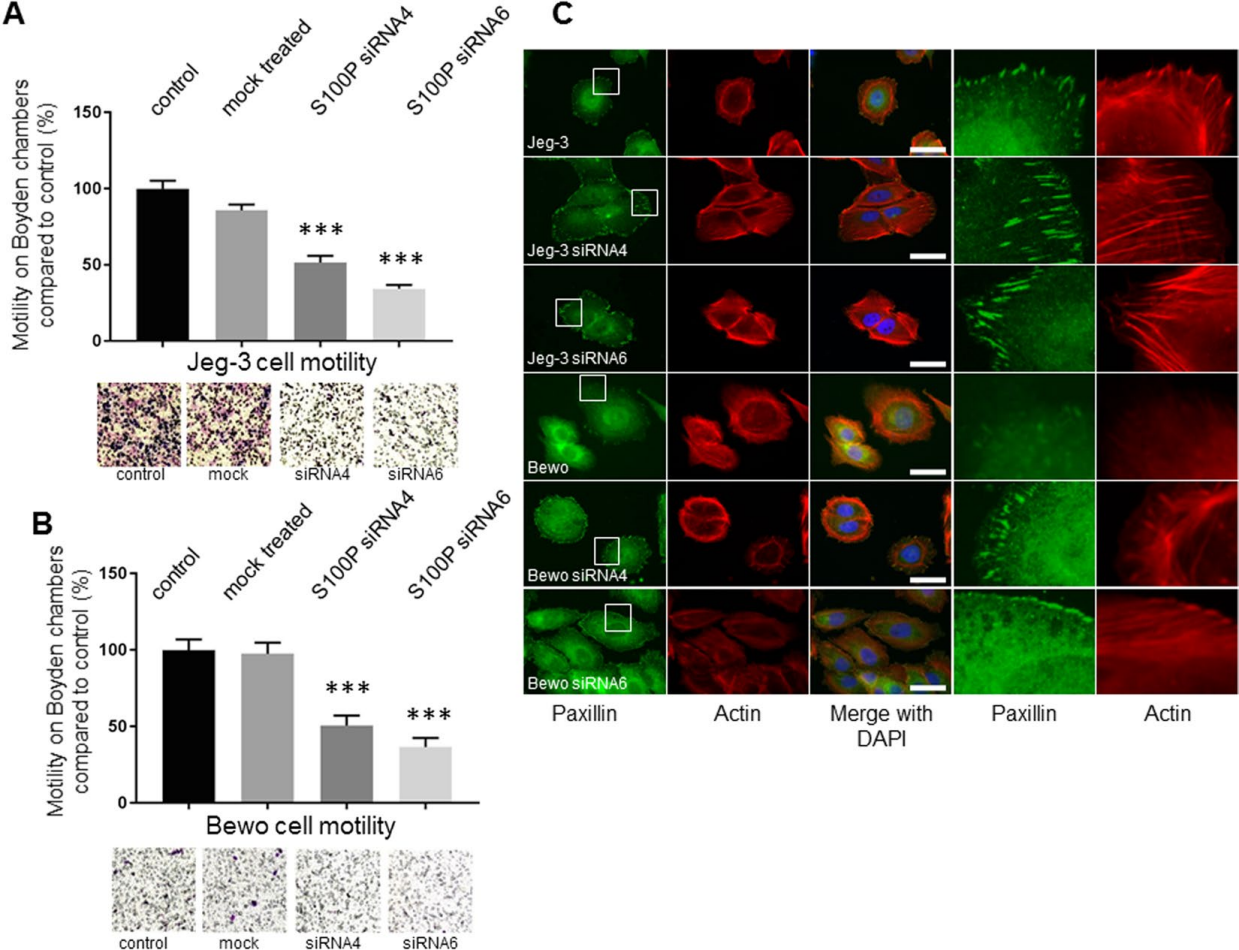
Specific reduction of S100P by siRNA technology leads to significant impairment in cellular motility of Jeg-3 and Bewo trophoblast cells. Bewo and Jeg-3 cells were treated with different S100P siRNAs (siRNA4 and siRNA6) or mock-control for 48 hours prior to starvation with low serum-containing medium. 24 hours later, cells were seeded into Boyden chambers for 16 hours prior to fixation and staining using the Diffquik histochemical kit for labelling of both nuclei and cytoplasm. (**A**,**B**) 5 random fields were quantified for each chamber. Data is presented as means ± SEM of 4 independent experiments relative to controls (percentage) from 4 replicate wells for each set of conditions. ***P < 0.0001 compared to control and mock treated (one way-ANOVA). After siRNA delivery, and a further 48 hours incubation, cells were seeded on fibronectin-coated coverslips and grown for a further 48 hours prior to fixation, permeabilisation and staining for the focal adhesion marker paxillin and the cytoskeletal marker actin. Cells were mounted and viewed using epifluorescence microscopy. (**C**) Images on the last row correspond to the focused regions of the highlighted cells. Bar corresponds to 50 μm. Images of representative fields of motility/invasion assays were taken with the EVOS XL Cell Imaging System at x20 magnification.

**Figure 5:**
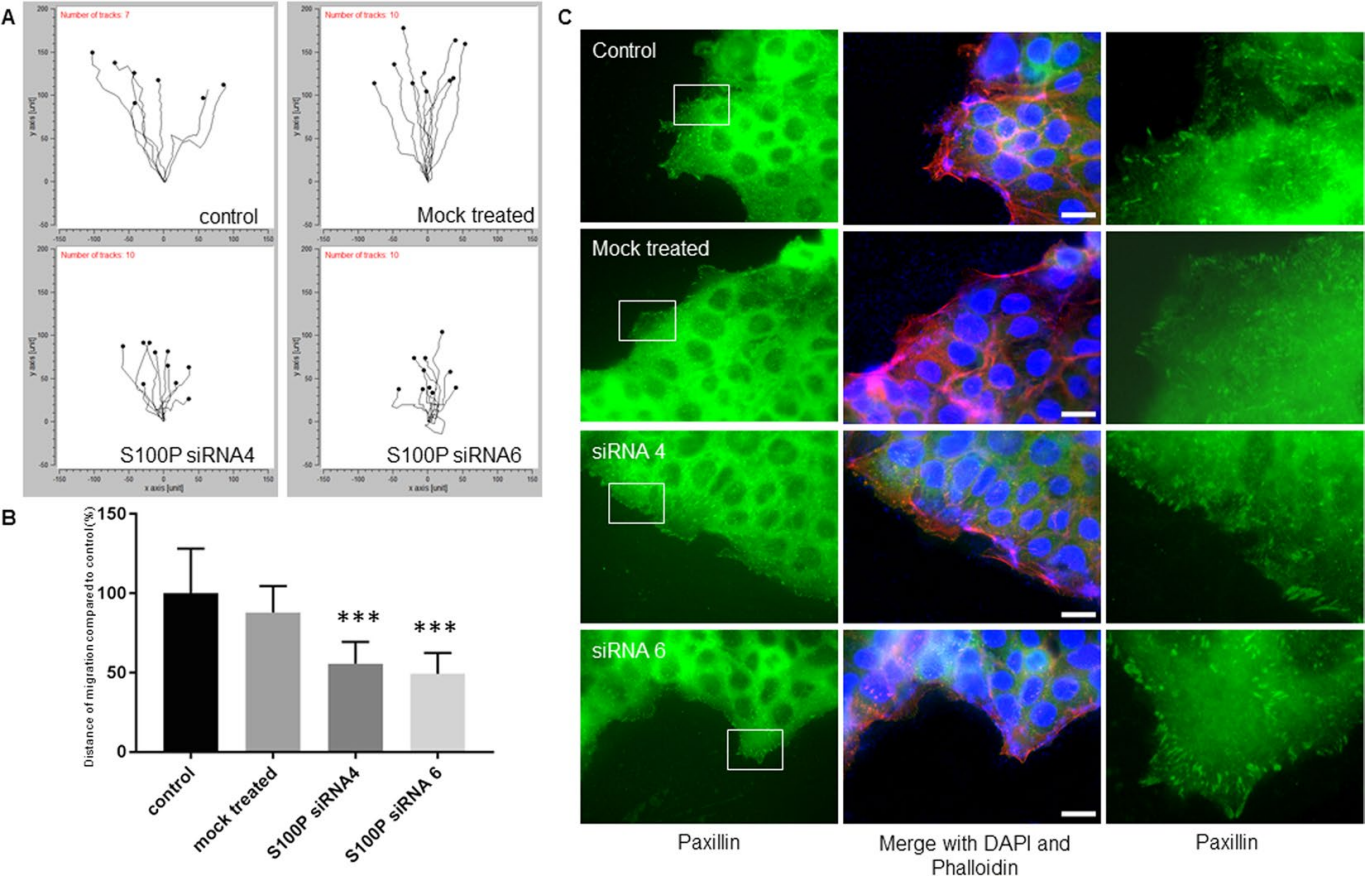
Specific reduction of S100P by siRNA leads to a reduction in distance of migration of Jeg-3 trophoblast cells. Jeg-3 cells treated with different S100P siRNAs (siRNA4 and siRNA6) or control mock for 48 hours were used either for quantitative chemotaxis-directed motility (**A**,**B**) or directional migration after wounding. (**C**) For chemotaxis, cells were first starved with low serum-containing medium for 24 hours prior to seeding on fibronectin coated coverslips in a Dunn chamber (**A**,**B**) against high serum containing medium. Digitised images were obtained using the Cell IQ automated image capture system, (Chip-Man Technologies) in which pre-selected fields were imaged using phase contrast microscopy on a continuous loop. Data collected was analysed using ImageJ and chemotaxis and migration software in order to analyse chemotaxis and distance of migration. Data is presented as mean values ± SD of 3 independent samples of a representative experiment compared to non-treated control samples. ***P < 0.0001 (one way-ANOVA). (**B**) For the wound healing experiment, cells were seeded on fibronectin-coated coverslips for 48 hours prior to attaining 100% confluency, scratched and grown for a further 16 hours prior to fixation, permeabilisation and staining for the focal adhesion marker paxillin and the cytoskeletal marker actin. Cells were mounted and viewed using epifluorescence microscopy. (**C**) Images in the last column correspond to the focused regions of the highlighted cells. Bar corresponds to 50 μm.

Since S100P can act as a regulator of cellular motility in multiple human cancer lines^23,31–33^, we sought to determine if S100P can similarly promote trophoblastic cell migration. Bewo and Jeg-3 cell migration (Fig. 4A,B) and chemotaxis (Fig. 5A,B) were monitored using Boyden and Dunn chamber assays, respectively, after S100P knock-down by siRNA delivery. Whilst mock treated samples demonstrated no significant changes in the number of cells able to migrate across the Boyden membranes (P = 0.068), Jeg-3 cells grown in the presence of siRNA4 or siRNA6 presented a significant reduction, by at least 50% (P < 0.0001), in the ability to do so (Fig. 4A). Treatment of Bewo cells with the same siRNAs resulted equally in significant reduction of motility (P < 0.0001; Fig. 4B). Thus, lowering the levels of S100P in trophoblast cells is responsible, at least in part, for a reduction in cell migration. Treatment of either Jeg-3 or Bewo cells with the other siRNAs also resulted in significant reduction in cell motility using this assay (Data not shown). By analogy with cancer cells^23^, these results suggest that S100P may affect cellular protrusions and/or cytoskeletal architecture. To decipher some of the underlying molecular mechanisms, we sought to stain components of focal adhesions and the actin cytoskeleton in control cells and in their S100P siRNA counterparts (Fig. 4C). Focal adhesion assembly and dynamics are key regulators of cellular motility, since the proteins complexed in these structures undergo profound remodelling over time^34^. One such protein, paxillin, has been shown to act as vital adapter that aggregates into the focal complex at early stages of their formation^35^. Punctate focal adhesion formation/maturation could be observed and characterised by their presence in small numbers and relatively punctate in size in the control cell lines. Upon incubation with siRNA4 and siRNA6, and a consequent reduction in S100P levels, clear increases in both the number and sizes of focal adhesions were observed (Table 1 and Fig. 4C). Quantitative analysis demonstrated a significant increase in numbers of focal adhesions by over 65% and 80% in Bewo and Jeg-3 cells respectively (Table 1) when cells were treated with either S100P siRNA4 or siRNA6 in comparison to control/mock-treated cells. Changes in actin cytoskeletal organisation were also seen when both cell lines were incubated with siRNA4 and siRNA6 (Fig. 4C). Untreated and mock control (data not shown) trophoblast cells demonstrated large arrangements of lamellipodia at the leading edges. Only small arrays of fibrillar actin could be observed and these rarely formed focal adhesions. Upon treatment with S100P siRNAs, large bundles of actin filaments could be seen at the cell periphery, resulting in reciprocal numbers of focal adhesion formations (Fig. 4C).

**Table 1:** Reduction of S100P in trophoblast cells leads to increases in the number of focal adhesions per cell.

Cell lines	Percentage focal adhesions per cell ± SEM (n = 50)	*P*-value^a^	*P*-value^b^
Jeg3 control	100 ± 2.22		
Jeg3 treated with S100P siRNA4	177.08 ± 4.54	P < 0.0001	
Jeg3 treated with S100P siRNA6	190.38 ± 3.67	P < 0.0001	0.4916
Bewo control	100 ± 1.85		
Bewo treated with S100P siRNA4	166.16 ± 3.04	P < 0.0001	
Bewo treated with S100P siRNA6	171.57 ± 3.99	P < 0.0001	0.9033

Jeg-3 and Bewo control cells, as well as cells treated with different S100P targeted siRNA for 72 hours were fixed and stained for paxillin and actin after seeding on fibronectin-coated coverslips. Data shown are means ± SEM corresponding to the average number of focal adhesion-containing paxillin observed per cell, presented as percentage of control. Untreated and mock treated controls (data not shown) were found to be not statistically significant (P > 0.05).

^a^P-value obtained from one-way ANOVA where total number of focal adhesions present in Jeg-3 or Bewo control cells were compared to previously S100P siRNA-treated counterparts.

^b^P-value obtained from one-way ANOVA where total number of focal adhesions present in Jeg-3 or Bewo cells treated with siRNA4 were compared to siRNA6-treated counterparts.

Because migration rates in Boyden chambers can be influenced by serum chemotaxis, we sought to determine whether the S100P-dependent changes in motility reported thus far were the result of increased motility or linked to the directional responsiveness of trophoblastic cells. To further this aim, directional cellular migration was observed in Jeg-3 cells by staining cells at the edge of a wound-healing scratch (Fig. 5C), or through their migration in Dunn chambers (Fig. 5A,B). When S100P expression in Jeg-3 cells was reduced, there was no significant effect on overall chemotaxis abilities, since all cells used, whether siRNA-treated or non-treated controls, all demonstrated efficient directional motility towards cues by moving in the same direction (Fig. 5A). By contrast, the presence of S100P was seen to have significant effects on the overall distances of cell migration (P < 0.0001; Fig. 5B). Thus whilst untreated or mock-treated samples were shown to have similar properties of migration rates, siRNA4 or siRNA6 delivery significantly reduced by 40%, the overall distance of migration of the Jeg-3 cells. These results are in line with the 50% decreases that had been observed when performing Boyden chamber assays.

To provide further information about the cellular changes taking place, we analysed components of focal adhesions in control cells and S100P siRNA-treated counterparts, in directionally migrating cells after scratch wounding (Fig. 5C). Once scratched, cells at the edge of the wound move in the newly created space, demonstrating a specific directional migration in the process^36–38^. After scratching, control and mock-treated trophoblasts established a small number of focal adhesion clusters, as determined by the formation of paxillin foci, at the cellular periphery (Fig. 5C and Table 2). However, when treated with either siRNA4 or siRNA6, a clear and significant increase of around 60% in the number of foci was seen per cell, suggesting that S100P downregulates the number and possibly maturation of focal adhesions at the leading edge of migrating cells.

**Table 2:** Reduction of S100P in trophoblast cells leads to increases in the number of focal adhesions per migrating cell.

Cell lines	Number of focal adhesions per cell ± SEM (n = 15)	*P*-value^a^	*P*-value^b^
Jeg-3 control	100 ± 1.98		
Jeg-3 mock control	106.13 ± 2.22	0.2858	
Jeg-3 treated with S100P siRNA4	160.65 ± 3.55	P < 0.0001	
Jeg-3 treated with S100P siRNA6	166.87 ± 3.06	P < 0.0001	0.9201

Jeg-3 control cells, as well as mock treated cells and cells incubated with different S100P targeted siRNA for 72 hours were grown until fully confluent prior to wound healing and grown for a further 16 hours before being fixed and stained for paxillin and actin. Data shown are means ± SEM corresponding to the average number of focal adhesions containing paxillin observed per cell at the leading front, presented as percentage of control. Untreated and mock treated controls (data not shown) were found to be not statistically significant (P > 0.05).

^a^P-value obtained from one-way ANOVA where total number of focal adhesions presents in Jeg-3 or Bewo control cells were compared to previously S100P siRNA-treated counterparts.

^b^P-value obtained from one-way ANOVA where total number of focal adhesions present in Jeg-3 or Bewo cells treated with siRNA4 were compared to siRNA6-treated counterparts.

High levels of S100P in trophoblasts have been linked to increased cell viability and cellular proliferation in the choriocarcinoma JAR cell line^19^. To verify whether the effects on cellular motility in the Jeg-3 and Bewo cells were related to reduced cell viability, we measured cell growth over 48 hours after S100P levels had been knocked down and therefore well within the time frame used to measure migration (Fig. 8A for Jeg-3 and data not shown for Bewo). None of the siRNA treatments resulted in a significant reduction of the cell numbers (P > 0.96 for all conditions tested) and hence cell viability was probably not compromised in our experiments as cells were proliferating normally over the course of the experiment, with a doubling of the population in about 24 hours indicating that the defects seen in motility are not due to reduction in cell numbers.

Taken together, this data demonstrates that the S100P protein can play an important role in the migratory rates of trophoblast cells and that reducing its expression levels results in a significant impairment in motility as well as affecting the overall actin cytoskeleton and the number of focal adhesions.

### Knock-down of S100P results in significant changes in Jeg-3 and Bewo cell invasion

**Figure 6:**
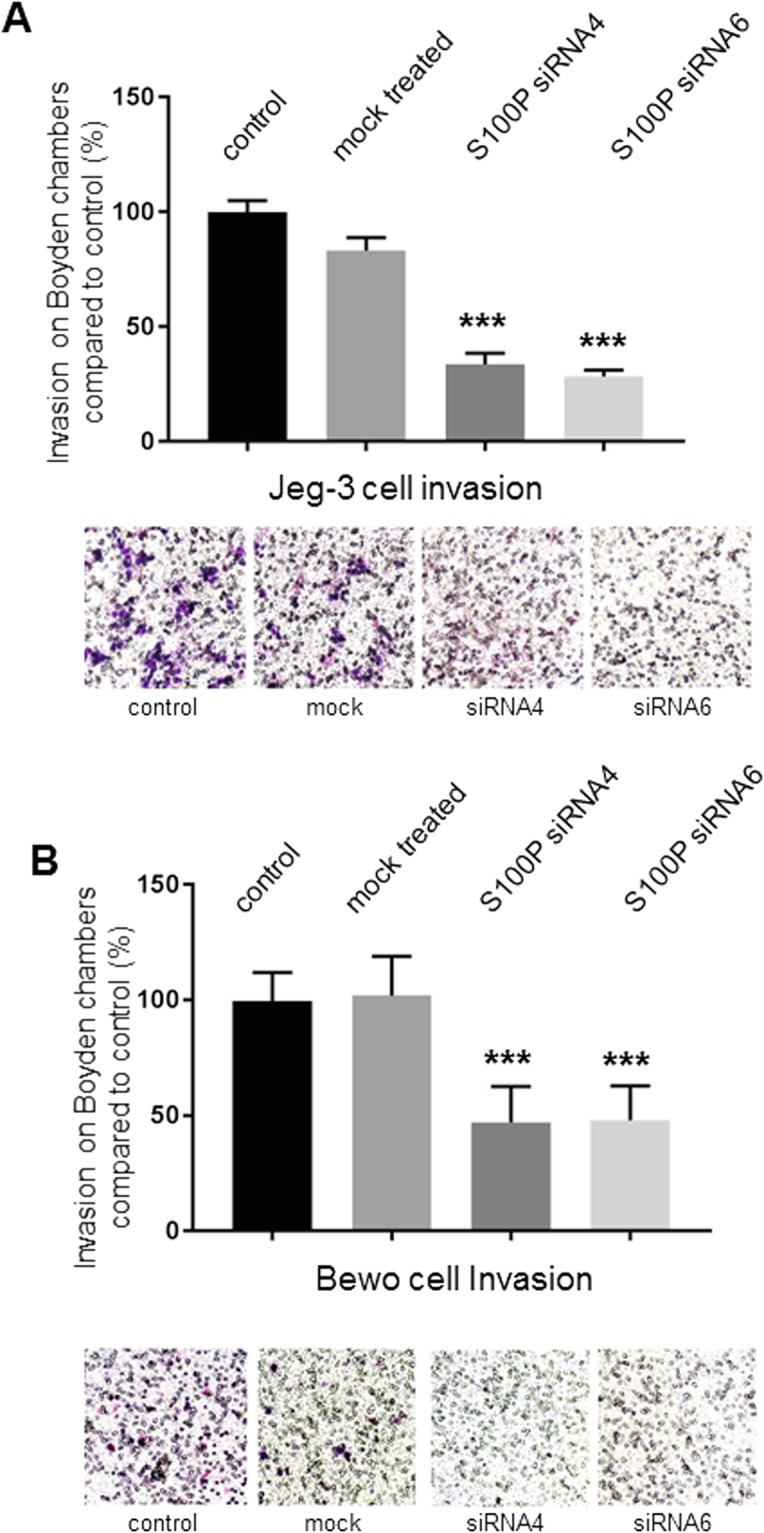
Specific reduction of S100P by siRNA leads to significant impairment in invasive abilities of Jeg-3 and Bewo trophoblast cells. Jeg-3 (**A**) and Bewo (**B**) cells were treated with different S100P siRNAs (siRNA4 and siRNA6) or control siRNAs for 48 hours prior to starvation with low serum containing medium. 24 hours later, cells were seeded in Boyden chambers previously coated with matrigel and incubated for 16 hours prior to fixation and staining using the Diffquik histochemical kit for labelling of both nuclei and cytoplasm. (**A**,**B**) 5 random fields were quantified for each chamber. Data is presented as means ± SEM of 4 independent experiments relative to controls (percentage) from 4 replicate wells for each set of conditions. ***P < 0.0001 compared to control and mock treated (one way- ANOVA). Images of representative fields of motility/invasion assays were taken with the EVOS XL Cell Imaging System at x20 magnification.

Given the well-established role for S100P in promoting cancer cell invasion, we assessed whether S100P could regulate the invasiveness of Jeg-3 and Bewo cells. Jeg-3 cells which had been previously treated for 48 hours with siRNA4 and siRNA6 targeted towards S100P, or the corresponding controls, were starved for serum for 24 hours prior to seeding on congealed matrigel in Boyden chambers and allowed to invade the gel in the membrane. The untransfected Jeg-3 cell controls as well as the mock-treated counterparts demonstrated good abilities to invade through Matrigel with no significant changes between them (Fig. 6). S100P siRNA treated Jeg-3 cells, however, showed a significant reduction in their ability to invade into the matrigel (P < 0.0001 compared to control or mock treated), lowering the number of cells capable of reaching the outer side of the membrane by more than 70% (Fig. 6A). Inhibition of invasion of Bewo cells using the same strategy was equally significant, causing a reduction of over 50–60% when treated with S100P directed siRNA compared to control counterparts (P < 0.0001; Fig. 6B).

All together the data demonstrates that reducing the expression of endogenous S100P in cultured Jeg-3 and Bewo trophoblastic cells causes them to decrease their ability to migrate, and more significantly, to invade.

### Overexpression of S100P in the first trimester extravillous HTR8/SVneo cell line leads to increased cellular motility and invasion

**Figure 7:**
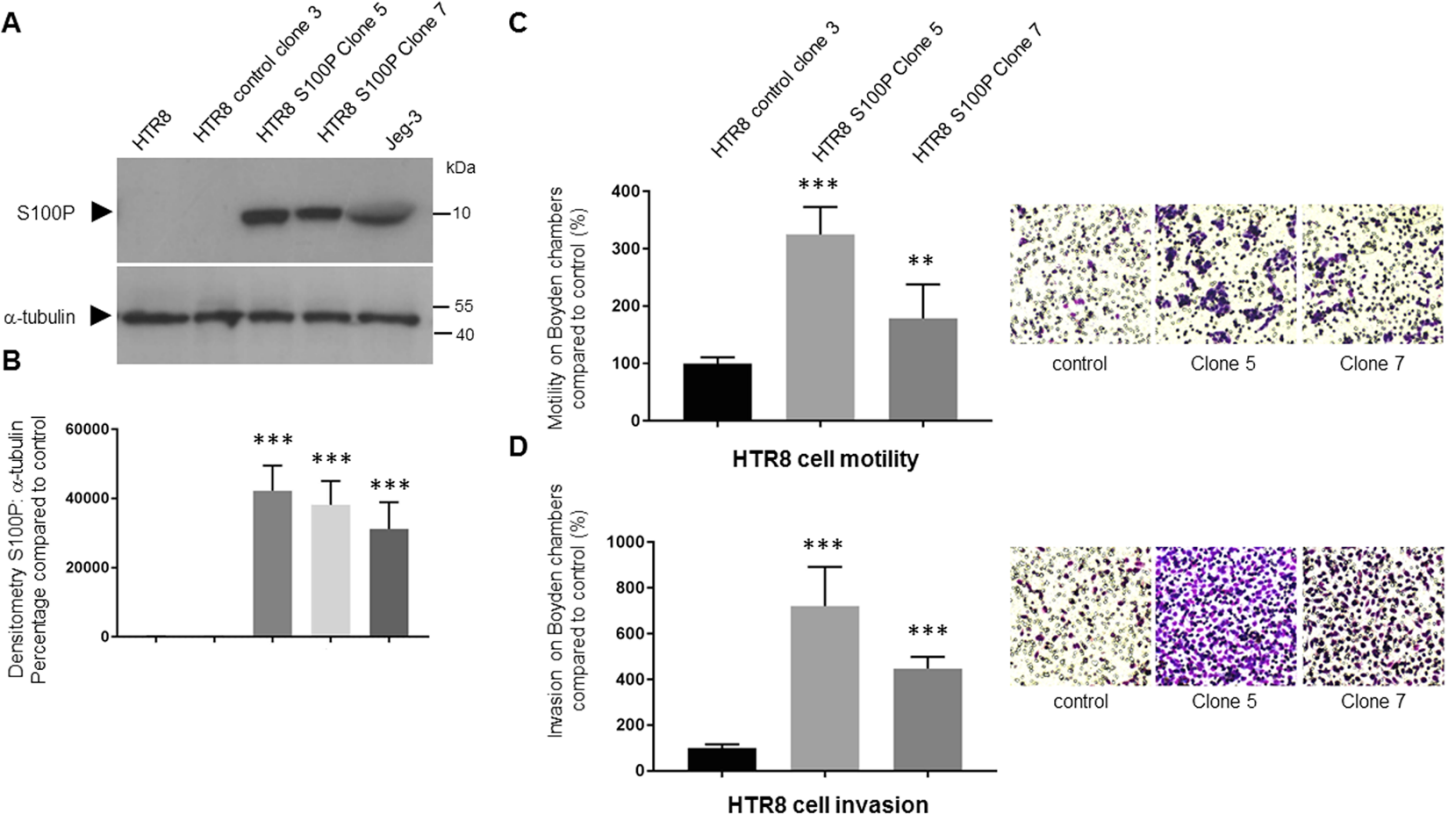
Overexpression of S100P in HTR8/SVneo trophoblast cells leads to significant increases in motility and invasive abilities. Stable transfection of HTR8/SVneo cells with S100P cDNA in pcDNA3.1 hygromycin plasmid was established to isolate clones expressing different levels of S100P, or their counterpart control, and protein levels assessed by Western blotting (**A**). Cells were collected and solubilised in Laemmeli buffer at equal loading and were separated by SDS-PAGE electrophoresis. Western blotting was carried out and membranes probed for S100P or α-tubulin and cropped images are presented (**A**). Expression levels of S100P were measured by densitometric analysis, normalised to α-tubulin and presented in comparison to the control untreated equivalent cells (**B**). Error bars in (**B**) show ± standard deviation compared to untreated control samples from a representative experiment. ***P < 0.001 compared to control (one way- ANOVA). The same clones expressing different levels of S100P were seeded in either Boyden chambers alone or chambers previously coated with matrigel. Cells were incubated for 16 hours prior to fixation and staining using the Diffquik histochemical kit for labelling of both nuclei and cytoplasm. 5 random fields were quantified for each chamber. Data is presented as a percentage compared to the control untreated cells for cell motility (**C**) or invasion (**D**). Error bars in (**C**,**D**) show means ± SD of 3 independent experiments relative to controls (percentage) from 4 replicate wells for each set of conditions. **P < 0.005 and ***P < 0.0001 compared to control cells (one way- ANOVA). Images of representative fields of motility/invasion assays were taken with the EVOS XL Cell Imaging System at x20 magnification.

To determine whether the S100P effects reported so far on cell migration and invasion can be reciprocated in a first trimester cell line, similar work was carried out using the well-established and characterised HTR8/SVneo cell line. Since S100P levels could not be detected in this line either by Western blotting or qPCR analysis (Fig. 2A; full length blots are presented in Supplementary Fig. S5; and 2B), stable transfected clones were engineered to overexpress S100P (Fig. 7A). The control clone 3 transfected with the empty backbone pcDNA3.1 hygromycin-B plasmid produced no detectable levels of S100P in contrast to both clones 5 and 7 transfected with the S100P containing plasmid SGB217 which demonstrated significant expression of the S100P protein, corresponding to a 20–40% increase over that of the Jeg-3 cells (Fig. 7A,B).

These high expressing clones were then used to determine the cells’ ability to migrate and invade using differential Boyden chamber assays. High levels of S100P expression in clones 5 and 7 resulted in significant increases in cellular motility and invasive traits (P < 0.0001). Whilst migration was significantly increased by about 100 and 250% for clone 7 and 5 respectively, compared to control clone 3, invasion was seen to be even more dramatically increased, with numbers reaching up to 5 fold for clone 7 and 8 fold for clone 5 respectively.

**Figure 8:**
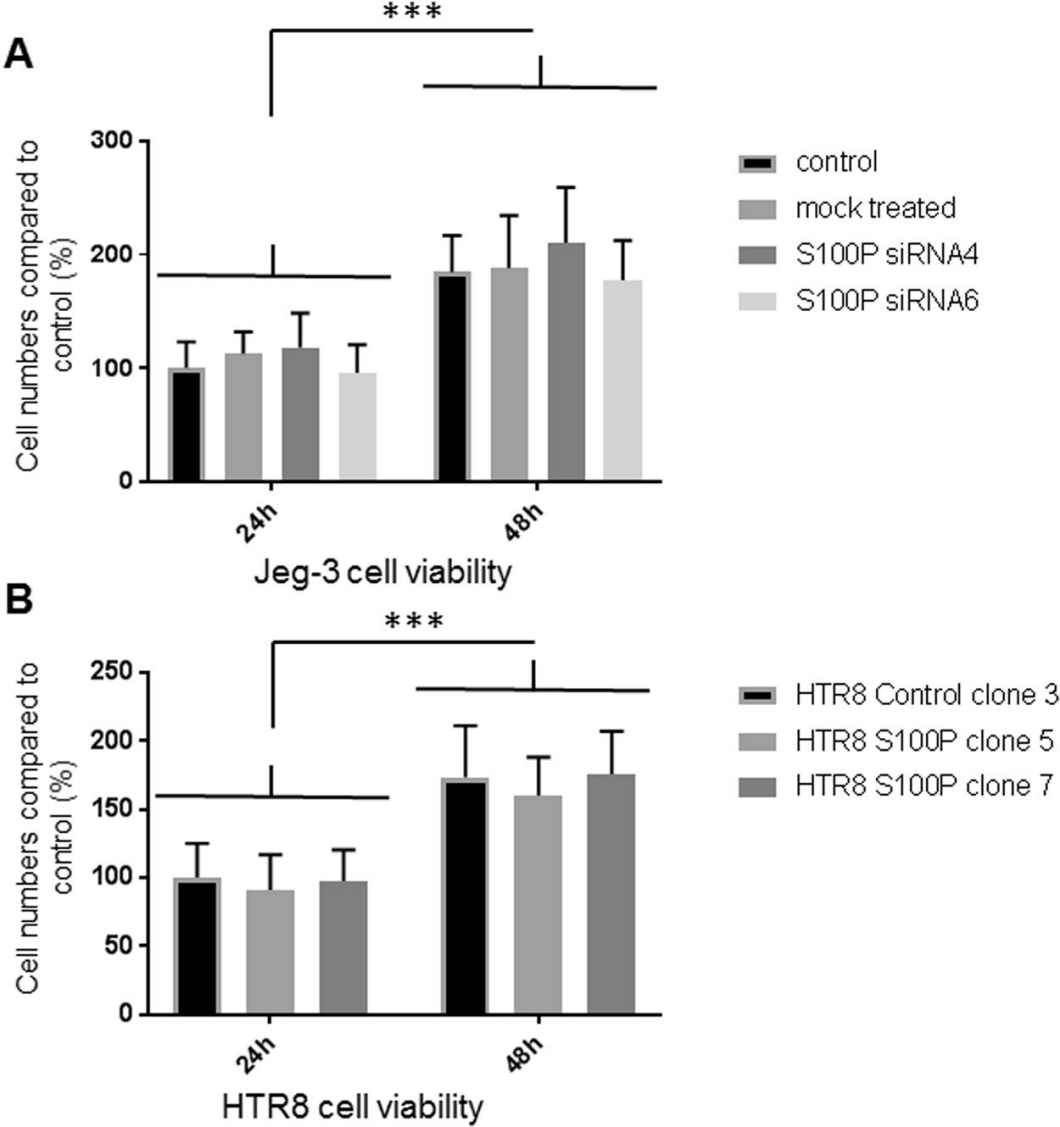
Modulation of S100P protein levels does not affect Jeg-3 and HTR8 trophoblast cell proliferation. Jeg-3 cells were treated with different S100P siRNAs (siRNA4 and siRNA6) or mock-control for 48 hours prior to seeding (**A**). Stably transfected HTR8/SVneo cells expressing S100P or the control counterpart were grown as described in methods (**B**). Cell lines were seeded into 24 well plates and left to grow for a further 24–48 hours before trypsinisation and removal from the wells and counting using trypan blue exclusion. Data is presented as percentage means ± SD of 3 independent experiments relative to controls from 3 replicate wells for each set of conditions. ***P < 0.0001 (one way-ANOVA).

Because regulating levels of S100P has been shown to affect viability and growth of the HTR8/SVneo cells^18^, we sought to determine whether high expression of S100P in these cells would affect cell numbers and therefore be responsible, at least in part, for the large effects reported here on cell motility and invasion. Despite the large excess in S100P expression seen in HTR8/SVneo expressing clones 5 and 7 compared to the control cells, we were not able to find any significant changes in cell proliferation and growth (P > 0.96 for all conditions tested) when assessing cell numbers over a 48-hour time, as cells were all seen to be growing normally with a doubling of the cell population about every 24 hours (Fig. 8B). These results demonstrate that overexpression of S100P significantly increases both motility and invasion in the first trimester extravillous HTR8/SVneo cell line.

## Discussion

S100P expression has been linked to progression of malignancy of cells originating from numerous tissue sources, such as breast, pancreas and lung^39–44^ with supporting evidence showing that S100P acts a regulator of cellular invasiveness and motility in multiple human carcinoma cell lines^23,31–33^. In this report, we offer the first evidence that expression of S100P can stimulate trophoblast cell motility and invasive properties, therefore supporting a new potential physiological role for S100P in the process of trophoblast implantation.

Recent reports suggest that S100P is expressed at high concentrations in trophoblast cells *in vitro* and *in vivo*^15,18,19,45^ with most results obtained in tissue culture as a result of global proteomic analysis^45^. Our work further supports the observation that S100P is actively expressed in trophoblast cells both in culture and *in vivo* (Figs 1 and 2). Furthermore, our analysis of S100P expression over the full gestation period demonstrates that its protein levels are highest during the early stage of implantation (first trimester), significantly reducing by 50% during the second and third trimester of gestation (Fig. 1). Such results corroborate, at least in part, those reported recently by Zhu *et al*.^19^ which also shows higher concentration of the protein in the early stages of placentation (first trimester) compared to the gestational second trimester period. However, whilst expression remained significantly lower during the final trimester of gestation in our work (Fig. 1), S100P expression in term placental tissues was seen to increase again in Zhu *et al*.^19^, suggesting potential functions of this protein during delivery. This observation shows that, during gestation, S100P levels peak in trophoblast cells when they are the most invasive^46^ and suggests that S100P could potentially be a regulator of this cellular process. In line with this reasoning, other proteins whose functions are essential during trophoblast invasion in implantation such as laminin α4 (LAMA4)^47^, placental protein 1 (PLAC1)^48^ and CUL1^49^ to cite just a few, have also been shown to be specifically highly expressed in the early stages of placentation. Similarly, other proteins of the S100 family such as S100A8 (MRP8) have also been found to be expressed in trophoblasts^12,50^ and its expression levels mirrored that of our work; that is, high levels found in the early stages of gestation with reducing levels as the pregnancy approached full term. The full implications of the expression of specific S100 proteins in early stages of trophoblast implantation needs to be explored further.

Immunohistochemistry analysis of the same tissues confirmed the reduction of S100P expression over time, whilst also clearly demonstrating the specific labelling of all trophoblasts (Fig. 1). In the first trimester, clear staining for S100P protein could be seen specifically in syncytiotrophoblasts as well as in cytotrophoblasts and extravillous trophoblasts in anchoring columns (Fig. 1E). In the latter, S100P expression colocalised with staining for HLA-G in the middle and distal extravillous populations *in vivo*. Whilst we have studied S100P as a stimulator of cellular motility and invasion in trophoblasts because of its presence in anchoring columns, it is clear that its expression in large amounts in the syncytiotrophoblasts suggests that it may confer other properties to placental development and may therefore be involved in events regulating the maternal-foetal interface. Further work is needed to explain other physiological functions of this protein during placentation.

Whilst all types of trophoblasts in tissues (Fig. 1.) as well as primary extravillous trophoblast cells isolated from first trimester placental tissues express high levels of S100P (Data not shown), no S100P was detected in the first trimester extravillous HTR8/SVneo cell line. Absence of S100P expression in this HTR background has recently been documented following global proteomic analysis^51^ whilst another group were able to detect its expression^18^. This discrepancy is difficult to explain, since we have been unable to get any positivity by either PCR or Western blotting, with quantitative analysis from both experiments demonstrating levels well below that of our negative control lines (Fig. 2). This could possibly be due to the fact that these cells have been obtained from two different sources, since S100P non-expressing HTR8/SVneo have been obtained directly from their creator (Graham Charles (Queen’s University, Kingston, Ontario (Canada)), whilst the others have been purchased from a cellbank. Whilst it is surprising that S100P was not found to be expressed in our HTR8 cell background, there is evidence of certain markers known to be present in extravillous trophoblast primary cultures and tissues which are not seen in this cell line. For example, expression of cytokeratin 7, HLA-G and E-cadherin have been reported as being absent in the HTR8/SVneo background, when in contrast, they are highly expressed in different trophoblast subtypes^52–56^. Similarly it is not uncommon for SVneo transformed cell lines to lose their abilities to express markers characteristic of their parental differentiated state when grown in tissue culture^57^. Furthermore, it has been shown that the use of matrigel is required to drive HTR8/SV neo cells into an EVT like cell phenotype^58,59^, regulating the expression of matrix metalloproteinases and integrin α1 for example. All experiments using the HTR8/SVneo cells performed in our work did not use matrigel, at least during studies related to their expression. Further work will be required to demonstrate if S100P can indeed be regulated as other genes considered to be markers of invasive EVT, when grown on matrigel.

Staining to establish the cellular localisation of endogenously expressed S100P demonstrated its presence in both the cytoplasm and nuclear domains of trophoblasts *in vivo* and in culture using the Jeg-3 and Bewo cell lines (Figs 1 and 2). Such observations have also been reported in other cells, both in tissues or lines, mainly in cancer states^23,41,60,61^ and demonstrated again with our HeLa cells (Fig. 2), suggesting that different pools of S100P may have differential functions, all which remain to be clearly identified and established both in cancer cells and in trophoblasts.

One such S100P dependent function that is widely accepted is its ability to promote cell migration and invasion^62^. In order to assess whether such properties could be affected in trophoblasts, the Jeg-3, Bewo and HTR8/SVneo cell lines were used as they provide a tractable experimental system recognised as an appropriate model for motile and invasive trophoblast behaviour^22,63,64^. We have now demonstrated here that S100P can play a pivotal role in regulating trophoblastic motility and invasion in cultured cells. Reducing S100P expression, especially in Jeg-3 cells, led to significant changes in migration distances (Fig. 5) and overall motility (Fig. 4), as well as a dramatic reduction in invasion (Fig. 6). Results obtained using Bewo cells also demonstrated clear defects in motility and invasion (Figs 4–6) but were somewhat reduced due to a less effective ability of delivered siRNAs to lower S100P expression (Fig. 3). This is in line with other work which has shown that siRNA treatment is less effective in Bewo cells compared to other trophoblast cell lines for targets such as tissue factor pathway inhibitor-2 (TFPI-2) or lasp-1^65,66^. In the reciprocal experiment using HTR8/SVneo cells, where no detectable amount of S100P could be seen, upregulating levels of S100P resulted in a dramatic improvement in both cell motility and invasion, with the latter being increased 10-fold in our higher expression clone. Modulation of gene expression improving both motility and invasion of the HTR8/SVneo cells has been reported, where for instance, expression of MSH homeobox 2^67^, CDX2^68^ and smurf2^69^ or the presence of Sphingosine-1-phosphate^70^ have been shown to increase invasion, albeit not to the levels that were seen in our experiments. This further demonstrates that despite the fact that S100P is not expressed in the HTR8/SVneo line, the molecular circuitry required for the S100P dependent pathway regulating both motility and invasion are present and further offer an invaluable model to further study the molecular mechanisms of such process.

This observation that S100P stimulates motility and invasion in trophoblast cells is consistent with some of the postulated roles for S100P in carcinoma cell lines^23,42,71–73^ as well as the process of metastasis in animals^72,73^, but as yet, has never been identified with a physiological process. In the context of this work, it highlights for the first time that S100P promotes migratory and invasive properties in human trophoblast cell lines and that, in line with its high expression during the first trimester in anchoring columns, further suggests a potential role for this protein in the process of implantation.

The clear mechanisms regulated by S100P to promote such changes in cellular motility and invasiveness are yet to be explained. Over the years, different target proteins such as ezrin^61^, IQGAP1^74^, non-muscle myosin IIA^23^ or proteins related to the tissue plasminogen activator pathway^75^ have been shown to act as S100P binding partners in normal and cancer cells where they are believed to regulate motility and invasion. Interestingly, ezrin knockdown has been shown to play key roles during gestation leading to foetal growth retardation^76^, whilst non-muscle myosin IIA is essential during embryogenesis and placentation^77^ and may regulate cellular motility of trophoblasts in culture^78^. However, a role for IQGAP1 and tissue plasminogen activator in trophoblast motility/invasion are yet to be characterised, but both have been shown to be key regulators of cancer motility/invasion^79–81^.

Our work here demonstrates that significant changes in the overall actin architecture takes place in trophoblasts when S100P levels are reduced. Less prominent motile features such as the formation of the leading edge can be observed (Figs 4 and 5). The actin cytoskeleton is a key regulator of cell migration and invasion, where the rearrangement of specific cellular structures known as lamellipodia and filopodia play an essential role^82^. Remodelling of the actin cytoskeleton has been closely linked to changes in cell motility in different cell lines as well as trophoblasts^83–85^. In this work, we show that reducing S100P levels via siRNA delivery leads to significant changes in the overall shape of the actin cytoskeleton, including the formation of large arrays of actin fibres at the cell periphery (Fig. 4) and the disappearance of lamellipodia spatial organisation. This concept is in line with previous observations that show that actin cytoskeletal integrity is critical for trophoblast differentiation and motility/invasion since highly motile undifferentiated trophoblast cells demonstrate prominent protrusions and less stress fibers/actin filament formation than their more differentiated counterparts in rat cells^86^. Equally, actin protrusion can be seen as a marker of cellular motility/invasion since treatment inhibiting LIMK was seen to both reduce overall actin remodelling at the leading edge and the general invasiveness of primary human cytotrophoblasts^87^. Whilst focal adhesion maturation and dynamics are key components of general cell motility, little is known about this process in trophoblasts. Motility of trophoblasts related to focal adhesion maturation is not well described, but reports have highlighted the importance of focal adhesion formation in trophoblasts^85,88–90^. We show here that reducing the levels of S100P by siRNA targeting in trophoblast cells results in a significant increase in the number and potential maturation of focal adhesions, given the clear changes in their number and size (Figs 4 and 5). Such observations are consistent with changes in cell motility, as more dynamics and less established focal adhesions have been demonstrated to be essential for migration, at least in carcinoma cell lines^25,91,92^ and also trophoblasts^86^. Similarly, changes in paxillin recruitment and focal adhesion kinase phosphorylation, both markers of focal adhesion maturation, have also been reported in trophoblasts^85,93,94^. The clear mechanisms by which S100P could remodel the actin architectures and focal adhesion assembly, as well as stimulate both cellular motility and invasion of trophoblasts, remain to be characterised.

## Electronic supplementary material

Supplementary legends for figures S1-S5 and tables S1-S3

Supplementary Figure S1-S5
