# Supplementary material for: S100P enhances the motility and invasion of human trophoblast cell lines
Source: Sci Rep. 2018 Jul 31;8:11488. doi: 10.1038/s41598-018-29852-2 (PMC6068119; doi:10.1038/s41598-018-29852-2)
Supplement: Supplementary file 1 — Supplementary legends for figures S1-S5 and tables S1-S3 [file 41598_2018_29852_MOESM1_ESM.docx]

**S100P enhances the motility and invasion of human trophoblast cell lines**

Maral E. A. Tabrizi, Tara Lancaster, Thamir M. Ismail, Athina Georgiadou, Ankana Ganguly, Jayna Mistry, Keqing Wang, Philip S. Rudland, Shakil Ahmad and Stephane R. Gross

**Supplementary Table S1**: Antibodies used in this study.

**Supplementary Table S2**: Total number of cells counted for motility and invasion of Jeg-3 or Bewo trophoblast cell lines after S100P siRNA delivery.

**Supplementary Table S3**: Total number of cells counted for motility and invasion of HTR8/SVneo trophoblast clones.

**Supplementary Table S1: Antibodies used in this study**

| **Antibody** | **Experiment** | | **Type** | **Clone** | **Supplier** | **Dilution** | **Incubation** | **Buffer** |  |  |  |
| --- | --- | --- | --- | --- | --- | --- | --- | --- | --- | --- | --- |
| **S100P** | IHC | Monoclonal | | IgG Rabbit | Abcam | 1/1500 | Overnight at 4⁰C | 1%BSA |  |  |  |
| **CK7** | IHC | Monoclonal | | Mouse | Leica Biosystems | 1/50 | Overnight at 4⁰C | 1%BSA |  |  |  |
| **HLA-G** | IHC | Monoclonal | | IgG1 Mouse | Abcam | 1/50 | Overnight at 4⁰C | 1%BSA |  |  |  |
| **CD49F** | IHC | Polyclonal | | IgG Rabbit | Abcam | 1/200 | Overnight at 4⁰C | 1%BSA |  |  |  |
| **Rabbit** | IHC | Biotinylated | | IgG Goat Anti-Rabbit | Vector Laboratories | 1/200 | 1hr room temperature | 1%BSA |  |  |  |
| **Mouse** | IHC | Biotinylated | | IgG Horse Anti-Mouse | Vector Laboratories | 1/200 | 1hr room temperature | 1%BSA |  |  |  |
| **S100P** | WB | Polyclonal | | Goat | R&D | 1/1000 | Overnight at 4⁰C | 3% BSA |  |  |  |
| **α-tubulin** | WB | Monoclonal | | Mouse | Sigma | 1/5000 | Overnight at 4⁰C | 3% BSA |  |  |  |
| **Goat HRP Secondary** | WB | Polyclonal | | Rabbit | Dako | 1/3000 | 2 hours at room temperature | 3% BSA |  |  |  |
| **Mouse-HRP Secondary** | WB | Monoclonal | | IgG Horse | Dako | 1/3000 | 2 hours at room temperature | 3% BSA |  |  |  |
| **S100P** | IF | Monoclonal | | IgG Rabbit | Abcam | 1/200 | 45 minutes at room temperature | 1% Goat serum |  |  |  |
| **Paxillin** | IF | Monoclonal | | Mouse | Invitrogen | 1/200 | 45 minutes at room temperature | 1% Goat serum |  |  |  |
| **α-Rabbit-FITC** | IF | Polyclonal | | Swine Polyclonal | Dako | 1/100 | 45 minutes at room temperature | 1% Goat serum |  |  |  |
| **α-Mouse-FITC** | IF | Polyclonal | | Rabbit Polyclonal | Dako | 1/100 | 45 minutes at room temperature | 1% Goat serum |  |  |  |

**Supplementary Table S2: Total number of cells counted for motility and invasion of Jeg-3 or Bewo trophoblast cell lines after S100P siRNA delivery.**

| Cell lines | Control | Mock treatment | siRNA 4 treated | | siRNA 6 treated |
| --- | --- | --- | --- | --- | --- |
| Jeg3 motility | 4203 | 3822 | | 2248 | 1562 |
| Jeg3 invasion | 414 | 333 | | 104 | 109 |
| Bewo motility | 994 | 949 | | 589 | 399 |
| Bewo invasion | 414 | 394 | | 183 | 181 |

Jeg-3 and Bewo control cells, as well as cells treated with different S100P targeted siRNA for 48h prior to seeding on Boyden chambers for motility or matrigel-coated Boyden chambers for invasion. Data presented in this Table are the sum of all cells counted on the far side of the chambers at the end of the incubation from four independent experiments (from Figures 4 and 6).

**Supplementary Table S3: Total number of cells counted for motility and invasion of HTR8/SVneo trophoblast clones.**

| Cell lines | Clone 3 control | Clone 5 S100P | Clone 7 S100P | |
| --- | --- | --- | --- | --- |
| HTR8/SVneo motility | 2168 | 6397 | | 3245 |
| HTR8/SVneo invasion | 906 | 7391 | | 4534 |

HTR8/SVneo control cells (clone 3), as well as cells expressing S100P (clones 5 and 7) were seeded on Boyden chambers for motility or matrigel-coated Boyden chambers for invasion. Data presented in this Table are the sum of all cells counted on the far side of the chambers at the end of the incubation from four independent experiments (from Figure 7).

**Supplementary Figure legends**

**Figure S1: Antibody specificity on serially sectioned human placenta samples**

Immunohistochemistry staining of serial human placental tissues using a panel of secondary antibodies and counterstaining were performed as described in Methods. Bar corresponds to 100 μm.

**Figure S2: S100P and α-tubulin protein levels in human placental samples at different gestation periods.** Expression of S100P proteins were analysed in both lysates obtained from different paraffin-embedded placental samples from different gestational periods (first trimester (n=5), second trimester (n=4), or third trimester (n=7)). Proteins extracted from paraffin-embedded placental block sections (1^st^ trimester; 2^nd^ trimester and 3^rd^ trimester) at equal loading were separated by SDS-PAGE electrophoresis. Western blotting was carried out and membranes probed for S100P or α-tubulin.

**Figure S3: S100P and α-tubulin protein levels in Jeg-3 and Bewo and HTR8 EV trophoblast cell lines.** HTR8, Bewo and Jeg-3 cells, along with HeLa A3 induced for S100P expression (or their non-expressing counterparts), were grown for 48 hours prior to collection for protein Western blotting after solubilisation in lysis buffer and Laemmeli buffer and equal loadings were separated by SDS-PAGE electrophoresis. Western blotting was carried out and membranes probed for S100P or α-tubulin.

**Figure S4: S100P and α-tubulin protein levels after specific knock-down in JEG-3 and Bewo trophoblastic cell lines.** Jeg-3 (**A**) and Bewo (**B**) cells were incubated in the presence of different S100P or control siRNAs for 48 hours and 72 hours prior to collection for protein Western blotting after solubilisation in lysis buffer and Laemmeli buffer and equal loadings were separated by SDS-PAGE electrophoresis. Western blotting was carried out and membranes probed for S100P or α-tubulin.

**Figure S5: S100P and α-tubulin protein levels in overexpressing HTR8/SVneo trophoblast cells.**

Stable transfection of HTR8/SVneo cells with S100P cDNA in pcDNA3.1 hygromycin plasmid was established to isolate clones expressing different levels of S100P, or their counterpart control, and protein levels were assessed by Western blotting. Cells were collected and solubilised in Laemmeli buffer and equal loadings were separated by SDS-PAGE electrophoresis. Western blotting was carried out and membranes probed for S100P or α-tubulin.
