# Supplementary material for: S100P enhances the motility and invasion of human trophoblast cell lines
Source: Sci Rep. 2018 Jul 31;8:11488. doi: 10.1038/s41598-018-29852-2 (PMC6068119; doi:10.1038/s41598-018-29852-2)
Supplement: Supplementary file 2 — Supplementary Figure S1-S5 [file 41598_2018_29852_MOESM2_ESM.pdf]

S100P enhances the motility and invasion of human trophoblast cell lines

Maral E. A. Tabrizi, Tara Lancaster, Thamir M. Ismail, Athina Georgiadou, Ankana Ganguly, Jayna Mistry, Keqing Wang, Philip S. Rudland, Shakil Ahmad and Stephane R. Gross

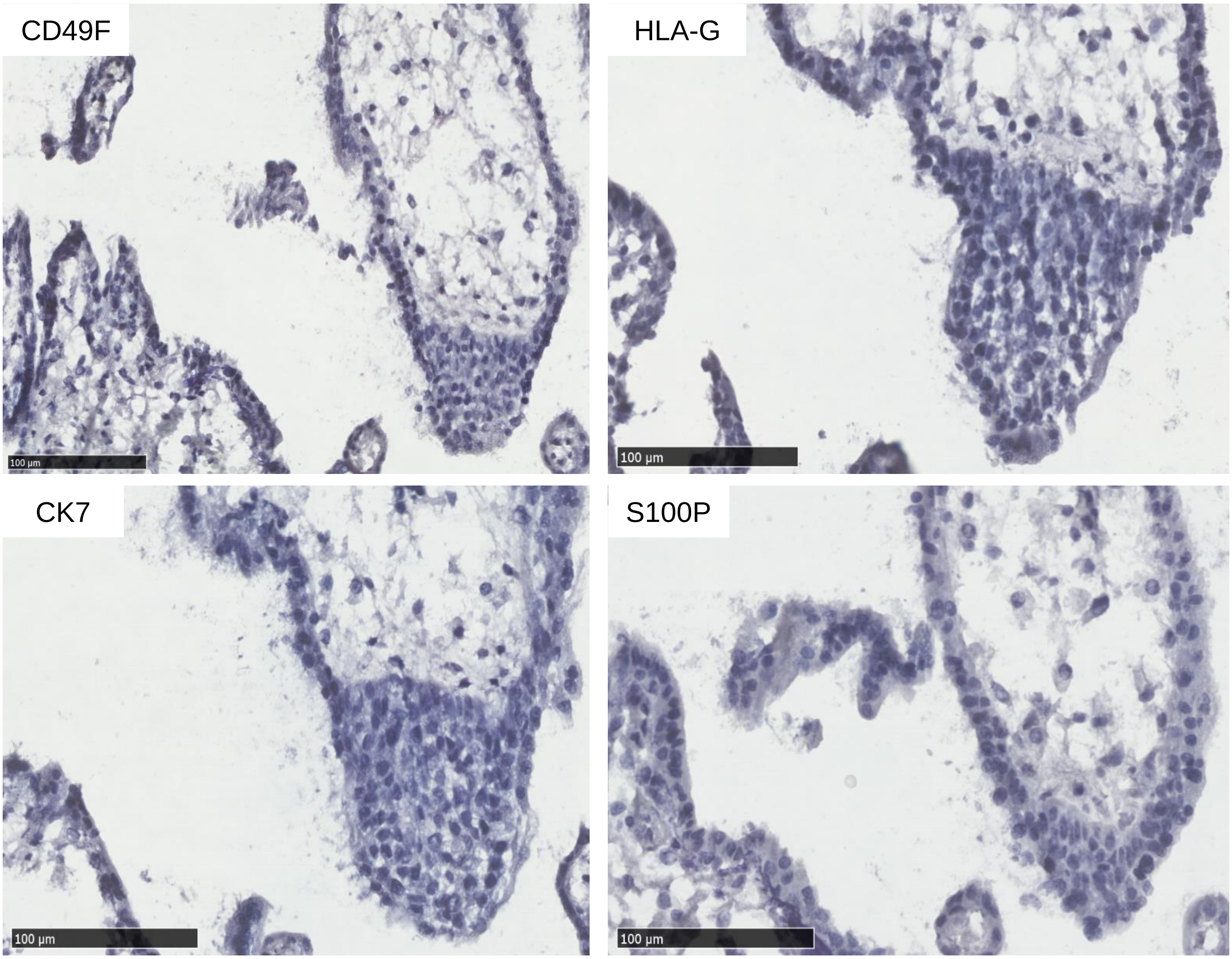

Figure S1

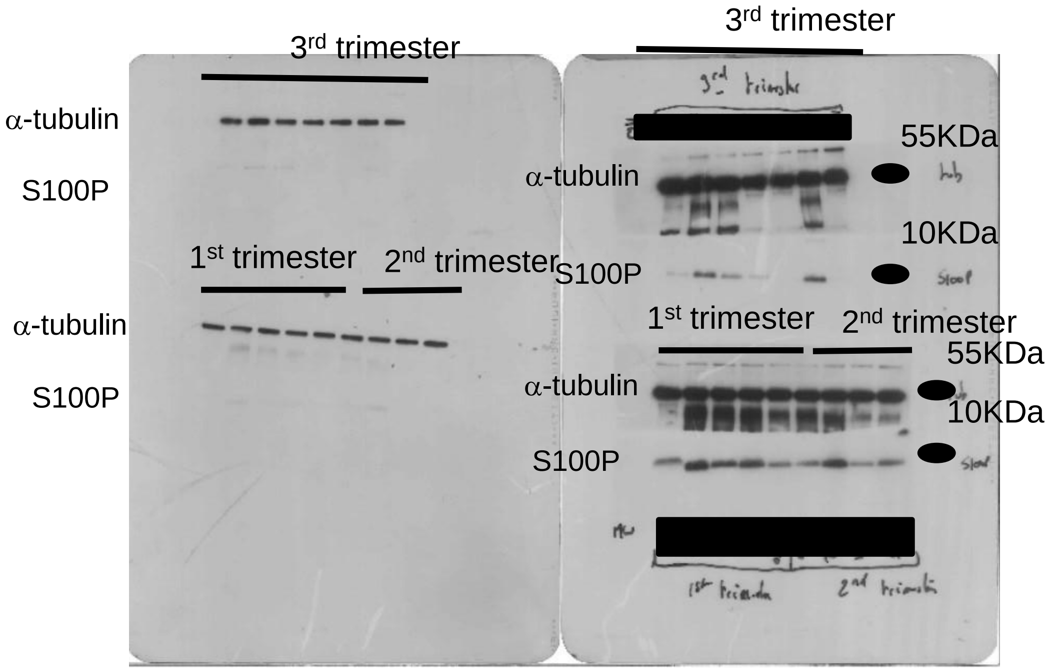

Figure S2

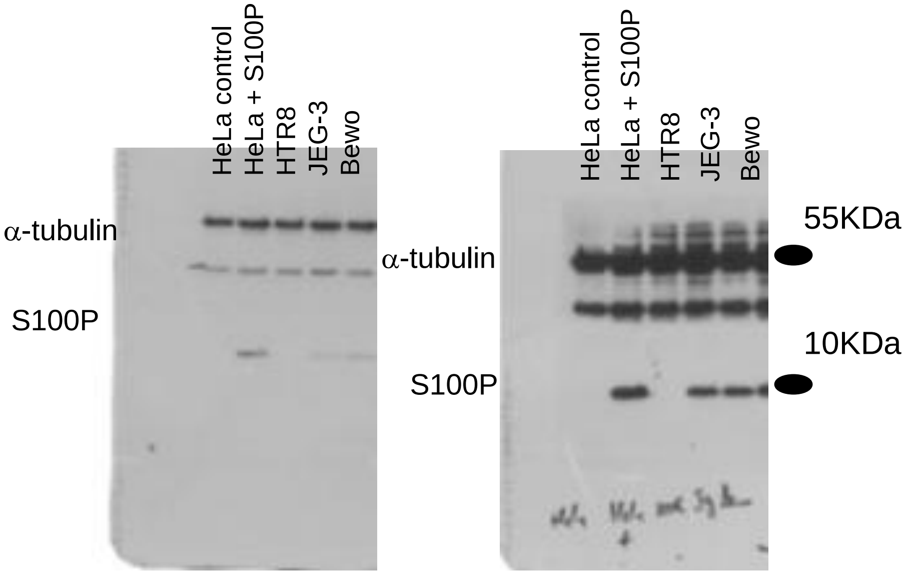

Figure S3

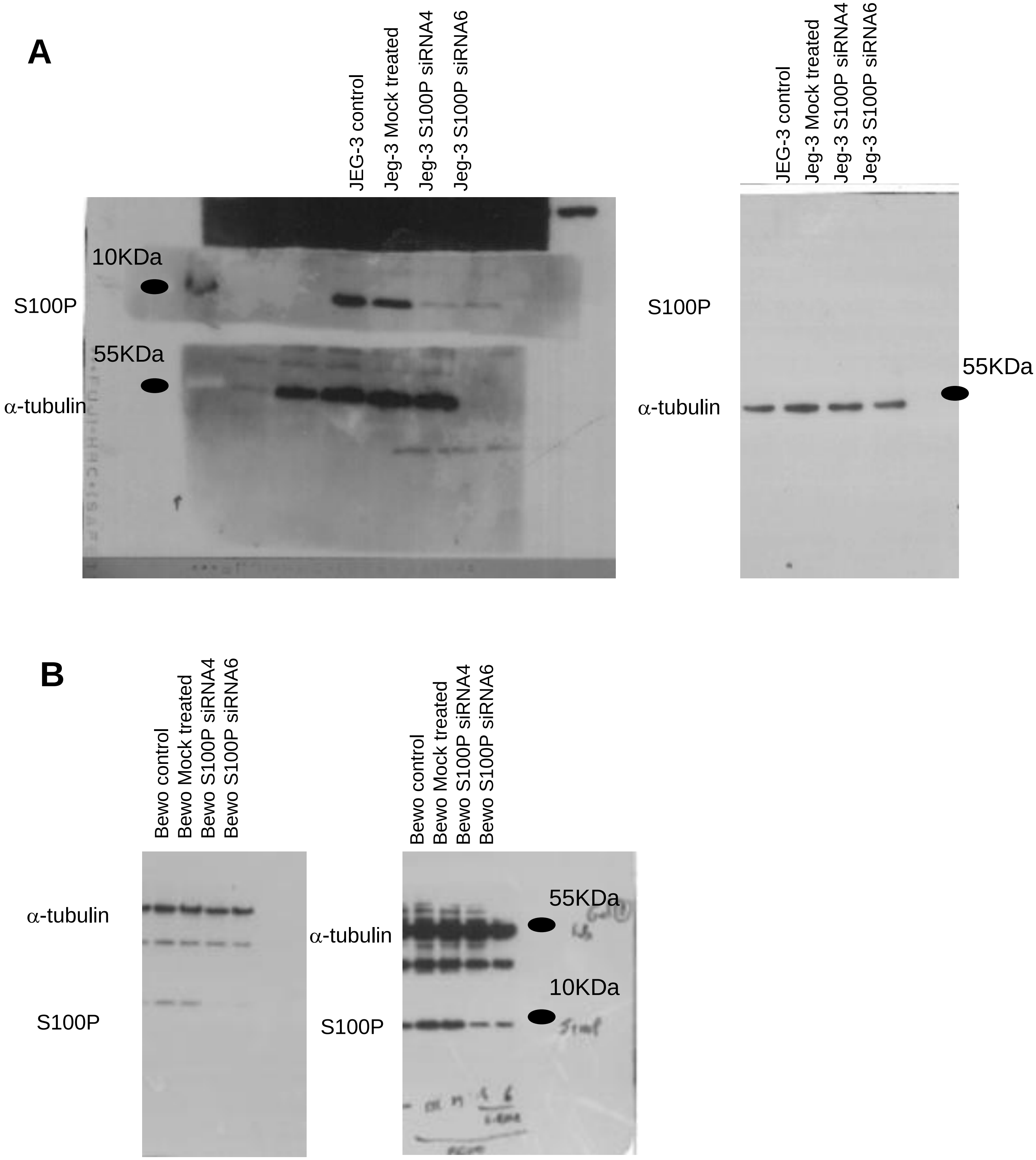
A
JEG-3 control
Jeg-3 Mock treated
Jeg-3 S100P siRNA4
Jeg-3 S100P siRNA6
10KDa
S100P
55KDa
α-tubulin
JEG-3 control
Jeg-3 Mock treated
Jeg-3 S100P siRNA4
Jeg-3 S100P siRNA6
S100P
55KDa
α-tubulin
B
Bewo control
Bewo Mock treated
Bewo S100P siRNA4
Bewo S100P siRNA6
α-tubulin
S100P
Bewo control
Bewo Mock treated
Bewo S100P siRNA4
Bewo S100P siRNA6
55KDa
α-tubulin
10KDa
S100P

Figure S4

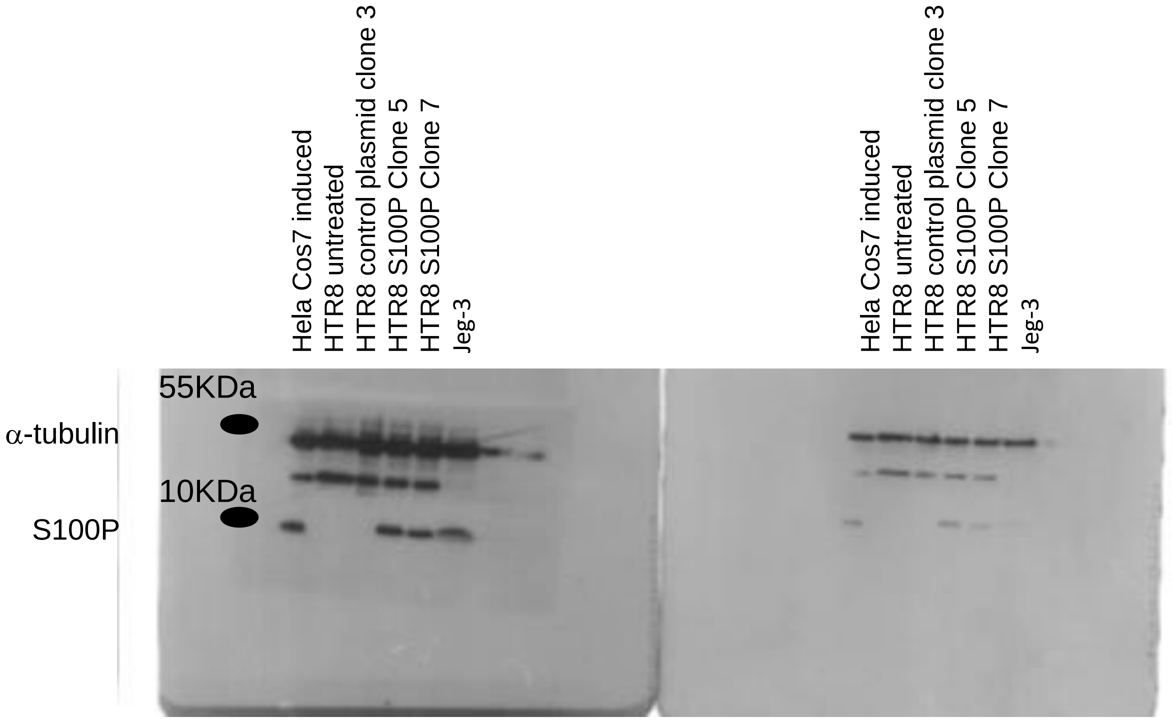
Hela Cos7 induced
HTR8 untreated
HTR8 control plasmid clone 3
HTR8 S100P Clone 5
HTR8 S100P Clone 7
Jeg-3
55KDa
α-tubulin
10KDa
S100P
Hela Cos7 induced
HTR8 untreated
HTR8 control plasmid clone 3
HTR8 S100P Clone 5
HTR8 S100P Clone 7
Jeg-3

Figure S5
